# Regularized impurity reduction: accurate decision trees with complexity guarantees

**DOI:** 10.1007/s10618-022-00884-7

**Published:** 2022-11-28

**Authors:** Guangyi Zhang, Aristides Gionis

**Affiliations:** Division of Theoretical Computer Science, KTH Royal Institute of Technology, Stockholm, Sweden

**Keywords:** Decision trees, Impurity functions, Submodularity, Tree complexity, Approximation algorithms

## Abstract

Decision trees are popular classification models, providing high accuracy and intuitive explanations. However, as the tree size grows the model interpretability deteriorates. Traditional tree-induction algorithms, such as C4.5 and CART, rely on impurity-reduction functions that promote the discriminative power of each split. Thus, although these traditional methods are accurate in practice, there has been no theoretical guarantee that they will produce small trees. In this paper, we justify the use of a general family of impurity functions, including the popular functions of entropy and Gini-index, in scenarios where small trees are desirable, by showing that a simple enhancement can equip them with complexity guarantees. We consider a general setting, where objects to be classified are drawn from an arbitrary probability distribution, classification can be binary or multi-class, and splitting tests are associated with non-uniform costs. As a measure of tree complexity, we adopt the expected cost to classify an object drawn from the input distribution, which, in the uniform-cost case, is the expected number of tests. We propose a tree-induction algorithm that gives a logarithmic approximation guarantee on the tree complexity. This approximation factor is tight up to a constant factor under mild assumptions. The algorithm recursively selects a test that maximizes a greedy criterion defined as a weighted sum of three components. The first two components encourage the selection of tests that improve the balance and the cost-efficiency of the tree, respectively, while the third impurity-reduction component encourages the selection of more discriminative tests. As shown in our empirical evaluation, compared to the original heuristics, the enhanced algorithms strike an excellent balance between predictive accuracy and tree complexity.

## Introduction

Decision trees are known to provide a good trade off between accuracy and interpretability. However, when their size grows, decision trees become harder to interpret, preventing their deployment in safety-critical applications and in domains where model transparency is highly valued, such as disease diagnosis. As interpretability still remains an ill-defined notion (Lipton 2018), in this paper we consider tree complexity, a commonly-accepted proxy, to quantify interpretability (Freitas 2014; Doshi-Velez and Kim 2017). In addition, low tree-complexity promotes cheaper and faster evaluation. Note that post-pruning techniques, such as the standard minimal cost-complexity pruning (Breiman et al. 1984), are heuristics performed mainly to avoid overfitting. Therefore, in order to produce interpretable trees, we aim for an integrated tree-induction algorithm that considers both the accuracy and complexity of the inferred trees.

**Fig. 1:**
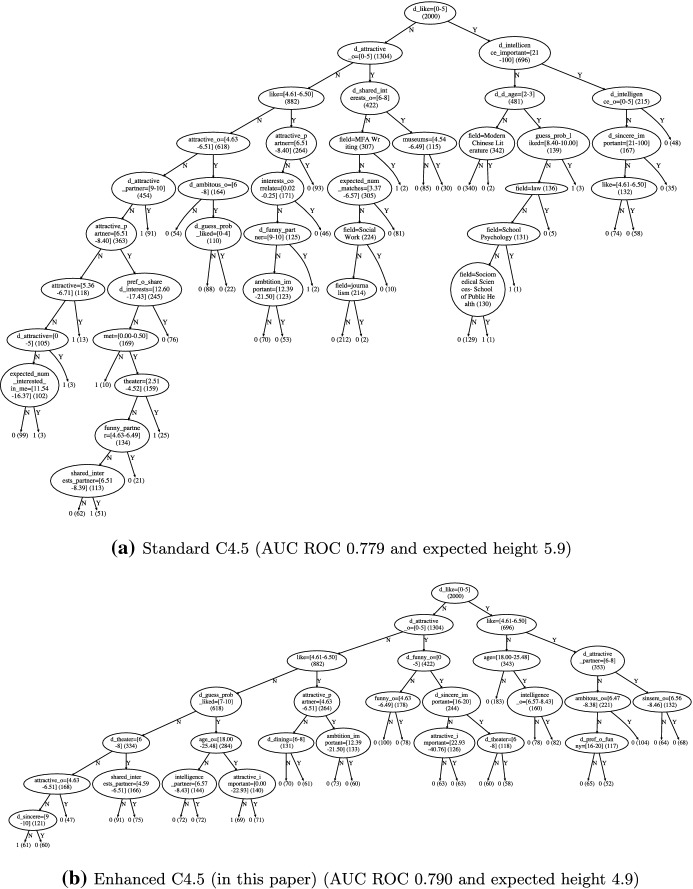
Decision trees for predicting if participants would like to see their date again after speed dating. Each internal node includes the test used and the number of participants in parentheses. Leaf nodes make a decision

More concretely, given a set of labeled objects (examples) drawn from an arbitrary probability distribution, our goal is to learn a decision tree that outputs the correct class of a given input object. Each internal tree node is equipped with a single test, e.g., a projection split along a feature, and each test is associated with a non-uniform cost, i.e., the cost of evaluating the outcome of the test. For example, an input object may represent a person, a test may correspond to a blood sugar test, and one possible outcome can be “high.” Our aim is to learn trees that are accurate and have low complexity. The latter complexity objective is measured by the expected cost to classify an object drawn from the input distribution; if all tests incur the same cost, this measure is simply the expected number of tests to classify an object. This complexity measure reflects a form of “local” interpretability: the more tests are involved in an if-then rule for a given object, the more obscure the rule becomes to a user (Freitas 2014). Figure 1 helps demonstrating this intuition by juxtaposing two decision trees with different complexity. Note that non-uniform test costs may arise in different real-world scenarios; for example, in a medical-diagnosis application some tests can be significantly more expensive than others.

The problem of minimizing the expected cost of a tree for perfect class identification has been extensively studied. Typically, the assumption of *realizability* (or consistency) is being made, which states that for every two distinct objects there exists at least one test that can distinguish them. Thus, one can always expand the tree until it classifies every object in the training data perfectly. Then, the goal is to find the tree with the minimum expected cost that classifies each object perfectly. Note that, in practice, the realizability assumption can be easily fulfilled by data preprocessing, as we demonstrate later in our experiments. When each object belongs to a distinct class, the problem is referred to as *entity identification* ($\hbox {EI}$) (Gupta et al. 2017). Without this restriction, the problem is called *group identification* ($\hbox {GI}$) (Cicalese et al. 2014). A further generalization that is called *adaptive submodular ranking* ($\hbox {ASR}$) (Navidi et al. 2020) characterizes the tree-building process as interaction among multiple submodular functions, one for each object, and achieves logarithmic approximation by a greedy algorithm. The above-mentioned works (Gupta et al. 2017; Cicalese et al. 2014; Navidi et al. 2020) consider the problem of building trees for the purposes of exact identification. They do not consider issues of accuracy and overfitting. In fact, exact identification on a set of (training) data leads precisely to overfitting.

The algorithm proposed for the $\hbox {ASR}$ problem by Navidi et al. (2020) provides a very elegant solution for the identification task it has been designed for. However, in practice, it is not suitable for classification tasks in the context of statistical learning, because the chosen tests are geared towards small expected cost and are not necessarily discriminative. Discriminative power is generally measured by the homogeneity of the target variable within a tree node, and is essential for the generalization of model performance over unseen data. Their method selects splits that minimize the number of *heterogeneous pairs* (also known as impure pairs) of objects (Golovin et al. 2010; Cicalese et al. 2014). In Section A we provide a simple example where the criterion favors a non-discriminative (presumably random) test over a discriminative one. While random tests lead to a balanced tree with bounded expected depth, they are not “learning”, that is, no statistical dependence is captured between tests and the target variable.

On the other hand, traditional decision-tree methods, such as $\hbox {CART}$  (Breiman et al. 1984) and $\hbox {C}4.5$  (Quinlan 1993), rely on time-tested impurity-reduction heuristics that yield decision trees with high discriminative power. Although trees produced by these popular methods are accurate in practice, there has been no guarantee on the size, or depth, of the resulting trees. Actually, despite the popularity of these methods, their theoretical properties remain still poorly understood (Bellala et al. 2012; Brutzkus et al. 2019; Blanc et al. 2020).

In this paper we propose a general family of methods that achieve the best of both worlds: *it produces decision trees having both high accuracy and bounded depth*. Our key discovery is that the $\hbox {ASR}$ framework can be extended to effectively analyze a broad range of impurity functions for tree induction.

More formally, we introduce the *non-overfitting group identification* ($\hbox {NGI}$) problem, which is a natural generalization of group identification ($\hbox {GI}$), where we further allow early termination during tree expansion to avoid overfitting. We propose a novel greedy algorithm that takes into consideration the impurity reduction and maintains the strong approximation guarantee on the complexity of the resulting tree. Specifically, our greedy algorithm admits the use of a *general family of decomposable impurity functions*, which is defined to be in the form of a weighted sum over impurity scores in each class. This family includes the popular functions of entropy and Gini-index. Therefore, our approach generalizes many traditional tree-induction algorithms such as $\hbox {CART}$ and $\hbox {C}4.5$ into a complexity-aware method.

In concrete, in this paper we make the following contributions.

- We extend the adaptive submodular ranking ($\hbox {ASR}$) framework of Navidi et al. (2020) and we propose a novel greedy algorithm to select discriminative tests for the non-overfitting group identification ($\hbox {NGI}$) problem. Our algorithm offers an asymptotically tight approximation guarantee on the complexity of the inferred tree under mild assumptions.
- We define a general family of decomposable impurity functions, which can be used by our algorithm as a surrogate for discriminative power. As a result, our algorithm generalizes traditional tree-induction algorithms, such as $\hbox {CART}$ and $\hbox {C}4.5$, into complexity-aware methods.
- We provide a comprehensive experimental evaluation in which we show that the enhanced $\hbox {C}4.5$ and $\hbox {CART}$ strike an excellent balance between predictive accuracy and tree complexity, compared to their corresponding original heuristics. Furthermore, the $\hbox {ASR}$ formulation yields inferior predictive accuracy, compared to other learning methods. Our implementation is publicly available.^1^

The rest of the paper is organized as follows. The related work is discussed in Sect. 2. The necessary notation and the formal definition of the $\hbox {NGI}$ problem are introduced in Sect. 3. The main algorithm and its theoretical analysis follow in Sects. 4 and 5, respectively. Empirical experiments are conducted in Sect. 6, and we conclude in Sect. 7.

## Related work

**Decision-tree induction.** Mainstream algorithms such as $\hbox {C}4.5$ and $\hbox {CART}$ embrace a top-down greedy approach. Most of the greedy criteria proposed are essentially ad-hoc heuristics for measuring the strength of dependence between tests and the class, with no consideration for tree complexity (Murthy 1998). Theoretical understanding about such greedy methods is still lacking in the literature. A lower bound on the expected tree depth for $\hbox {C}4.5$ that depends on the shape of a given tree has been developed by Bellala et al. (2012). There also exist some recent results in the field of learning theory (Brutzkus et al. 2019; Blanc et al. 2020).

**Tree complexity** Popular measures include the number of nodes in the tree, the tree height and the expected path length. The first kind of measures are closer to a notion of “global” interpretability, in the sense that one could inspect the entire tree of a small size, while the second kind of measures provide a notion of “local” interpretability, in the sense that one could explain any given object using a small number of tests. Our choice in the paper, the third kind of a measure, combines elements from both global and local interpretability. First, it obviously enables a form of local interpretability, i.e., a guarantee of a small expected number of tests when explaining a given object. This choice is considered to be more natural and less strict compared to worst-case tree height, as it may not be possible to classify every object using a small number of tests. Second, it also enables a form of global interpretability, as the global model knowledge is acquired by understanding the decision for every example in the dataset, and also it leads to smaller trees in general. Unfortunately, these complexity measures are proven to lead to $\textbf{NP}$-hard tasks (Hancock et al. 1996; Laurent and Rivest 1976). In particular, the expected path-length measure with an arbitrary probability distribution over objects does not admit sub-logarithmic approximation (Chakaravarthy et al. 2007).

**Identification** The entity identification ($\hbox {EI}$) problem has been investigated in different contexts, including optimal decision trees, disease/fault diagnosis, and active learning (Adler and Heeringa 2008; Chakaravarthy et al. 2007; Dasgupta 2005; Garey 1972; Guillory and Bilmes 2009; Gupta et al. 2017; Kosaraju et al. 1999). A class-based generalization, the group identification ($\hbox {GI}$) problem, where objects are partitioned into groups (classes), has also been studied (Bellala et al. 2012; Cicalese et al. 2014; Golovin et al. 2010). The state-of-the-art method achieves $\mathcal {O} (\log n)$-approximation in a general setting with an arbitrary object distribution and non-uniform multi-way testing costs (Cicalese et al. 2014). Our paper further generalizes the latter work by considering the discriminative power of the selected tests. To the best of our knowledge, this is the first work to combine identification problems and traditional tree-induction algorithms.

**Stochastic submodular coverage** (${\textsc {StoSC}}$) Tree induction can be seen as a sample-based stochastic submodular-coverage problem (Golovin and Krause 2011; Grammel et al. 2016), by relating a realization of items in the ${\textsc {StoSC}}$ problem to an object in identification problems. The expected cost of a tree is then equivalent to the expected cost in item selection.

**Adaptive submodular ranking (ASR)** The $\hbox {ASR}$ problem, proposed by Navidi et al. (2020), originates from the line of research of min-sum set cover (Feige et al. 2004; Im et al. 2012), and turns out to generalize the above-mentioned identification problems (Bellala et al. 2012; Cicalese et al. 2014; Golovin et al. 2010). Our formulation follows the framework of $\hbox {ASR}$, and extends its greedy criterion to incorporate an impurity-reduction component.

## Problem definition

In this section, we first formalize the *non-overfitting group identification* ($\hbox {NGI}$) problem, and then define a family of decomposable impurity functions for tree induction.

An instance of the $$\hbox {NGI}$$ problem is specified by a set of objects $$X =\{x _1,...,x _{n} \}$$, a set of class labels $$L =\{\ell _1,...,\ell _{k} \}$$, and a set of tests $$D =\{d _1,...,d _{m} \}$$. The objects in $$X$$ are drawn from a probability distribution $${p}$$, i.e., object $$x$$ in $$X$$ occurs with probability $${p} (x)$$. Each object $$x \in X $$ is associated with a class $$\ell (x)$$ in $$L $$. A test $$d \in D $$ performed on an object $$x \in X $$ returns a value $$d (x) \in \{1,...,\nu _d \}$$. We assume that employing test $$d$$ incurs cost $${c} (d)$$. For simplicity and without loss of generality, we also assume that the cost function $${c}$$ takes integral values. A useful quantity in our later analysis is the minimum object probability $$p_{\text {min}} =\min _{x \in X} {p} (x)$$. Finally, we assume that a threshold parameter $$\theta \in [0,1]$$ is given as input, which determines a stopping condition for the decision-tree construction, as we will see shortly.

We write ${T} (X)$ to refer to a decision tree built to classify the objects in $X$. We omit the reference to the set $X$ when it is clear from the context and just write ${T}$. We also write ${T} (S)$ to refer to a subtree of the decision tree to classify objects in a node $S$ of the tree, where $S \subseteq X$ is the subset of objects. Each internal node $S$ is equipped with a test $d$ in $D$. Objects in $S$ are partitioned by test $d$ into multiple subnodes according to their testing outcomes $d (x)$. Using this convention we refer to the root of the decision tree simply as $X$, that is, the complete set of objects to be classified by the tree. Finally, we define ${p} (S)=\sum _{x \in S} {p} (x)$.

We stop splitting a node $S \subseteq X$ in the tree ${T}$ when either (*i*) the node $S$ is *homogeneous*, i.e., all objects in $S$ belong to the same class, or (*ii*) the probability ${p} (S)$ is no greater than the threshold parameter $\theta$, for instance, in the case of uniform ${p}$, the node $S$ has at most $\theta n$ objects. As a surrogate for homogeneity, we adopt a function $\varvec{\phi }$ over pairs of objects. We define $\varvec{\phi } (S)$ to be the number of *heterogeneous* pairs of objects in the node $S$, i.e., pairs of objects with distinct classes. Note that $\varvec{\phi } (S)=0$ when $S$ is homogeneous.

As a measure of complexity for a tree rooted at $X$, we adopt the measure of *expected cost*, which we denote by ${c} ({T} (X))$. In particular, we define ${c} ({T},x)$ as the cost of evaluating an object $x$ in ${T}$, which is the sum of costs of all tests that $x$ goes through in ${T}$. The expected cost of a tree ${T}$ for a set of objects $X$ is then defined as ${c} ({T} (X)) = \sum _{x \in X} {p} (x) \, {c} ({T},x)$.

We are now ready to define the $\hbox {NGI}$ problem.

### Problem 1

(*Non-overfitting group identification (NCI)*) Given a problem instance $I = (X, L,D,\ell ,{p}, {c}, \theta )$, with set of objects $X$, set of class labels $L$, set of tests $D$, object labels $\ell$, probability distribution ${p}$, cost function ${c}$, and a threshold $\theta$, find a tree ${T} (X)$ that minimizes the expected cost ${c} ({T} (X))$ and for all leaf nodes $S$ it satisfies either $\varvec{\phi } (S)=0$ or ${p} (S)\le \theta$.

The $\hbox {NGI}$ problem generalizes the $\hbox {GI}$ problem by setting $\theta =0$, and as stated in Sect. 2, the $\hbox {GI}$ problem is $\textbf{NP}$-hard. Thus, we aim to find a tree ${T}$ that is an approximate solution, i.e., whose cost ${c} ({T})$ is bounded with respect to the cost ${c} ({{{T}}^{*}})$ of the optimal tree ${{{T}}^{*}}$.

Our approach draws inspiration from the *adaptive submodular ranking* ($\hbox {ASR}$) problem (Navidi et al. 2020), which can be defined similarly, by replacing each object $x _i$ in $X$ with a non-decreasing submodular function $f _i: 2^D \rightarrow [0,1]$ such that $f _i(\emptyset )=0$ and $f _i(D)=1$; recall that $D$ is the set of tests, and thus, each function $f _i$ takes as input a subset of tests. We denote the set of non-decreasing submodular functions by $F = \{f _i\mid x _i \in X \}$. We again consider a tree, which recursively partitions $F$. The tests $D$ and the probability distribution ${p}$ apply to the set of functions $F$ in the same way that they apply to their corresponding objects. For example, a function $f _i$ evaluated on a test $d \in D$ returns a value $d (f _i)=d (x _i)$, which determines the branch of the tree that $f _i$ will follow. Given a tree ${T}$, a function $f$ picks up all tests associated with the nodes it goes through and is *fully covered* when it reaches its maximum function value $f (D)$. Let ${c} ({T},f)$ be the cost of *covering* $f$ in ${T}$, defined as the sum of costs of all tests that $f$ goes through in ${T}$ before it is *fully covered*. Note that a function is not necessarily covered in a leaf node, it may be covered in an internal node. The expected cost of a tree ${T}$ is defined in a similar manner as for the $\hbox {NGI}$ problem. The *adaptive submodular ranking* problem is defined as follows.

### Problem 2

(*Adaptive submodular ranking (ASR)*  (Navidi et al. 2020)) Given a problem instance $I = (F, D, {p}, {c})$, with set of submodular functions $F$, set of tests $D$, a probability distribution ${p}$, and cost function ${c}$, find a tree ${T} (F)$ that covers all functions in $F$ and minimizes the expected cost ${c} ({T} (F)) = \sum _{f \in F} {p} (f) {c} ({T},f)$.

**Decomposable impurity functions** When constructing decision trees for classification tasks, in addition to having small expected cost, the discriminative power of the selected tests is also vital. A number of different impurity measures have been widely used in deciding a discriminative test in decision trees, such as *entropy* and *Gini index*. Such impurity measures are defined as functions $h: [0,1]^k \rightarrow \mathbb {R}_{+}$, taking as input the class distribution at a given tree node. Impurity functions are expected to satisfy certain conditions (Kearns and Mansour 1999), which capture the notion of “impurity.” All impurity functions mentioned in this paper satisfy the following conditions: (1) they obtain the maximum value if the class distribution is uniform, and the minimum value zero if a node is pure (i.e., homogeneous); (2) they are concave; and (3) they are symmetric.

A typical splitting criterion compares the change in impurity before and after performing a test $d$, defined as $h (S)$ and $h (S \mid d)$, respectively. The *impurity reduction* of a test $d$ on a tree node $S$ is defined as $d (S,d) = h (S) - h (S \mid d)$. A test that causes larger impurity reduction is considered more discriminative. Based on the concavity property of $h$, it is easy to show that $d (S,d)\ge 0$ for any tree node $S$ and test $d$. We defer the proof of this claim to the Appendix, Section B.

Before we define a special family of impurity functions for our problem, we first introduce some additional notation. For a node $$S$$ of the tree, where $$S \subseteq X $$, we define $$S ^v _d $$ as the child node of $$S$$ by equipping $$S$$ with test $$d$$ and following the branch that takes on a specific testing value $$v $$. In particular, we define $$S ^{(i)}_d = S ^{v =d (x _i)}_d $$. Likewise, we define $$S ^{(i)}_{{D}^{\prime }} $$ as the ending node of a path that starts at $$S \subseteq X $$ and follows a sequence of nodes each equipped with a test $$d$$ in $${{D}^{\prime }} \subseteq D $$ by taking on a value of $$d (x _i)$$. Note that the order of tests in $${{D}^{\prime }}$$ does not matter in $$S ^{(i)}_{{D}^{\prime }} $$. Finally, we denote the total probability of objects in a specific class $$\ell $$ in $$S$$ as $${p} _\ell (S)=\sum _{x \in S:\ell (x)=\ell } {p} (x)$$.

We are now ready to define $h (S)$ and $h (S \mid d)$ for our problem. We require $h$ to be *decomposable*, i.e., to be a weighted sum over impurity scores in each class. We define:

1 $$\begin{aligned} h (S) ~=~ \sum _{\ell } \frac{{p} _\ell (S)}{{p} (S)} h _\ell (S) ~=~ \frac{1}{{p} (S)} \sum _{x \in S} {p} (x) h _{\ell (x)}(S), \end{aligned}$$

where $h _\ell (S)$ can be any function of $\frac{{p} _\ell (S)}{{p} (S)}$, the proportion of objects of class $\ell$ in $S$, which ensures that $h$ satisfies the three requirements stated above (i.e., (1) being maximized at uniform class distribution and minimized at homogeneity, (2) concavity, and (3) symmetry).

A wide range of concave impurity functions adopt such a form. For example, $h$ becomes the entropy function when $h _\ell (S) = -\log \frac{{p} _{\ell }(S)}{{p} (S)}$, and it becomes the Gini index when $h _\ell (S) = 1-\frac{{p} _{\ell }(S)}{{p} (S)}$. With the impurity of a node $S$ defined, $h (S \mid d)$ is just a weighted sum of the impurity of all child nodes of $S$ when split by test $d$, i.e., $h (S \mid d) = \sum _{v \in [\nu _d ]} \frac{{p} (S _d ^v)}{{p} (S)} h (S _d ^v)$.

A useful quantity for our analysis is the maximum value of $h _{\ell (x)}(S)$, which we denote by $\epsilon _h =\max _{S \subseteq X} \max _{x \in S} h _{\ell (x)}(S)$.

## Algorithm

The main idea of our approach is to cast the $\hbox {NGI}$ problem as an instance of the $\hbox {ASR}$ problem (Navidi et al. 2020). We achieve this by defining a non-decreasing submodular function for each object. The $\hbox {ASR}$ problem is solved by a greedy algorithm that picks tests to maximize the coverage of the submodular functions while encouraging a balanced partition. We further incorporate the impurity-reduction objective into the greedy criterion to encourage the selection of discriminative tests, without losing the approximation guarantee.
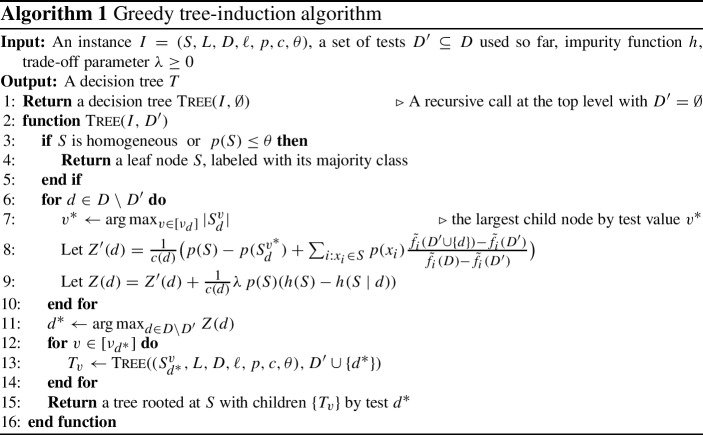


Our algorithm for the $\hbox {NGI}$ problem is demonstrated in Algorithm 1. It is a greedy algorithm, which, at each node $S$, selects a test $d$ that maximizes a cost-benefit greedy score $Z (d)$ consisting of the following three terms:

2 $$\begin{aligned} Z (d) ~=~ \frac{1}{{c} (d)} \left( \underbrace{ B (d) }_{\text {balance}} + \underbrace{ E (d) }_{\text {efficiency}} + \,\,\lambda \!\!\!\! \underbrace{ D (d) }_{\text {discrimination}} \right) . \end{aligned}$$

The first term, $B (d) = {p} (S) - {p} (S _d ^{{{v}^{*}}})$, with ${{v}^{*}} = \arg \max _{v \in [\nu _d ]} |S _d ^v |$, is the sum of the branch probabilities except the largest-cardinality branch. Maximizing $B (d)$ encourages selecting a test $d$ that yields a balanced split.

The second term,

$$\begin{aligned} E (d) = \sum _{i:x _i\in S} {p} (x _i) \frac{\tilde{f} _i({{D}^{\prime }} \cup \{d \}) - \tilde{f} _i({{D}^{\prime }})}{\tilde{f} _i(D) - \tilde{f} _i({{D}^{\prime }})}, \end{aligned}$$

is the re-weighted total sum of the marginal gain in each submodular function, which we will define for our objects shortly. Maximizing $E (d)$ accelerates the progress towards termination.

The last term, $D (d) = {p} (S) \left( h (S) - h (S \mid d)\right)$, is the impurity reduction we defined in Sect. 3, which improves the discrimination of the selected test. The user-defined parameter $\lambda \ge 0$ controls the trade-off between tree complexity and discrimination.

One way to understand the greedy score $$Z (d)$$ is to view the $$B $$ and $$E $$ terms as a *regularizer*. Notice that maximizing only the first two terms, $${{Z}^{\prime }}(d) = \frac{1}{{c} (d)} \left( B (d)+E (d)\right) $$ at Step 8 of Algorithm 1, is exactly the greedy criterion used by Navidi et al. (2020) to solve the $$\hbox {ASR}$$ problem.

We finish the description of our method by showing how to define the submodular function $$f _i$$ for each object $$x _i$$. We start by defining two monotonically non-decreasing submodular functions. For each object $$x _i\in X $$, both submodular functions take as input a subset of tests $${{D}^{\prime }} \subseteq D $$ and return a real value. The first function $$f^{{p}} _i({{D}^{\prime }})$$ is defined as the scaled total probability of the objects that do not fall into $$S ^{(i)}_{{D}^{\prime }} $$, i.e., the objects that disagree with $$x _i$$ in at least one test in $${{D}^{\prime }} \subseteq D $$. Note that eventually, only object $$x _i$$ itself stays in $$S ^{(i)}_D $$. Formally, we define $$f^{{p}} _i$$ as

$$\begin{aligned} f^{{p}} _i({{D}^{\prime }}) = (1 - {p} (S ^{(i)}_{{D}^{\prime }})) / (1-{p} (x _i)). \end{aligned}$$

The second function $$f^{\varvec{\phi }} _i({{D}^{\prime }})$$ is defined as the number of heterogeneous pairs that do not fall into $$S ^{(i)}_{{D}^{\prime }} $$. Eventually, no heterogeneous pair will exist in $$S ^{(i)}_D $$ and the ending node is homogeneous. We define

$$\begin{aligned} f^{\varvec{\phi }} _{i}({{D}^{\prime }}) = (\varvec{\phi } (X) - \varvec{\phi } (S ^{(i)}_{{{D}^{\prime }}})) / \varvec{\phi } (X). \end{aligned}$$

The target (maximum) values for these two functions are both 1, for each object $x _i$. Thus, the functions $f^{{p}} _i$ and $f^{\varvec{\phi }} _{i}$ are *fully covered* for a subset of tests ${{D}^{\prime }} \subseteq D$ for which $f^{{p}} _i({{D}^{\prime }}) = 1$ and $f^{\varvec{\phi }} _{i}({{D}^{\prime }}) = 1$, respectively. It is easy to see that both functions are submodular and monotonically non-decreasing. When the termination constraint $\theta$ for minimum probability is in place, we use the fact that the monotonicity and submodularity properties remain valid when truncated by a constant. The truncated version of the $f^{{p}} _i$ function is defined as

$$\begin{aligned} \bar{f^{{p}} _i}({{D}^{\prime }}) = \min \left\{ (1 - {p} (S ^{(i)}_{{D}^{\prime }})) / (1-\max \{{p} (x _i),\theta \}), 1\right\} . \end{aligned}$$

Next we define the *disjunction* function $\tilde{f} _i$ of $\bar{f^{{p}} _i}$ and $f^{\varvec{\phi }} _i$, which remains monotonically non-decreasing and submodular (Deshpande et al. 2016; Guillory and Bilmes 2011). We set

$$\begin{aligned} \tilde{f} _i({{D}^{\prime }}) = 1 - \left( 1 - \bar{f^{{p}} _i}({{D}^{\prime }})\right) \left( 1 - f^{\varvec{\phi }} _i({{D}^{\prime }})\right) . \end{aligned}$$

It is easy to see that with a reasonable value of $\theta$ (e.g., a multiple of the greatest common divisor of $\{ {p} (x) \}$), the minimum positive incremental value of any element and any $\tilde{f} _i$ is

$$\begin{aligned} \Delta = \min _{\begin{array}{c} i \in [n ],\,{{D}^{\prime }} \subseteq D, \\ d \in D: \tilde{f} _i({{D}^{\prime }} \cup \{d \}) > \tilde{f} _i({{D}^{\prime }}) \end{array}} \left\{ \tilde{f} _i({{D}^{\prime }} \cup \{d \}) - \tilde{f} _i({{D}^{\prime }}) \right\} = \Omega (p_{\text {min}}/n ^2). \end{aligned}$$

Last, we examine the time complexity of Algorithm 1. The greedy score for a tree node $$S$$ with respect to a test can be computed in $$\mathcal {O} (|S |)$$ time. At each level of the decision tree, the union of disjoint nodes has a total size $$n$$. Thus, the worst-case time complexity is $$\mathcal {O} (H m n)$$, where $$m $$ is the number of tests, $$n $$ is the number of objects, and *H* is the tree height, which is upper bounded by $$n $$. In practice, the algorithm is more efficient than what this worst-case bound suggests; it has the same time complexity as standard tree-induction algorithms, such as $$\hbox {CART}$$.

## Approximation guarantee

In this section we establish the approximation guarantee of Algorithm 1 for the $\hbox {NGI}$ problem. Our main result is the following.

### Theorem 1

Algorithm 1 provides an $\mathcal {O} (\log 1/p_{\text {min}} + \log n + \lambda \epsilon _h )$ approximation guarantee for the $\hbox {NGI}$ problem.

As a practical consequence of Theorem 1, we have the following corollary, which is a consequence of the fact that for the popular impurity functions the factor $\epsilon _h$ in the approximation ratio of Theorem 1 can be effectively bounded. Note that in practice $p_{\text {min}} \ge 1/n$ when given training data of $n$ points, and thus $\mathcal {O} (\log 1/p_{\text {min}})=\mathcal {O} (\log n)$. We omit the constant $\lambda$ for simplicity.

### Corollary 2

Algorithm 1 provides an $$\mathcal {O} (\log 1/p_{\text {min}} + \log n)$$ approximation guarantee for the $$\hbox {NGI}$$ problem when the impurity function $$h $$ is either the entropy or the Gini index function.

### Proof

In addition to the result of Theorem 1 we can show that $$\epsilon _h $$ is small, compared to the other terms, or bounded by a constant. When $$h $$ is the entropy function, we have

$$\begin{aligned} \epsilon _h&= \max _{S \subseteq X} \max _{x \in S} \left\{ h _{\ell (x)}(S) \right\} = \max _{S \subseteq X} \max _{x \in S} \left\{ -\log \frac{{p} _{\ell (x)}(S)}{{p} (S)} \right\} \\&\le -\log \frac{\min _{x \in X} {p} (x)}{{p} (X)} = \log 1/p_{\text {min}}. \end{aligned}$$

When $$h $$ is the Gini index function, we have

$$\begin{aligned} \epsilon _h&= \max _{S \subseteq X} \max _{x \in S} \left\{ h _{\ell (x)}(S) \right\} = \max _{S \subseteq X} \max _{x \in S} \left\{ 1-\frac{{p} _{\ell (x)}(S)}{{p} (S)} \right\} \le 1. \end{aligned}$$

$$\square $$

Assuming $$\textbf{P} \ne \textbf{NP} $$, the result given by Theorem 1 is asymptotically the best possible among instances where $$1/p_{\text {min}} $$ is polynomial in $$n $$. This follows directly from the hardness result of the $$\hbox {EI}$$ problem (Chakaravarthy et al. 2007), which in turn, is proved via a reduction from the minimum set-cover problem. Recall that by specifying $$\theta $$ to be zero, $$\hbox {NGI}$$ problem degenerates into $$\hbox {EI}$$ or $$\hbox {GI}$$ problems. The constructed $$\hbox {EI}$$ instance in their reduction asks for a minimum object probability $$p_{\text {min}}$$ such that $$1/p_{\text {min}} =\Theta (n ^3)$$, and thus if $$\hbox {NGI}$$ admits $$o (\log 1/p_{\text {min}}) = o (\log n)$$ approximation, we could solve the set-cover problem with $$o (\log n)$$-approximation, which is conditionally impossible (Feige 1998).

### Remark 1

The $\hbox {NGI}$ problem does not admit an $o (\log n)$ approximation algorithm, unless $\textbf{P} = \textbf{NP}$.

### Proof of Theorem 1

Our analysis is similar to the one by Navidi et al. (2020), except that we need a new proof of their key lemma for our new greedy selection rule (Eq. (2)). This is done by leveraging the special structure in the family of impurity functions (Eq. (1)) we employ.

In order to analyze the total cost along a path, we treat cost as discrete “time” — or continuous time if we allow continuous cost — and we divide time geometrically. We refer to the decision tree returned by Algorithm 1 as ${T} _{\text {A}}$, while we refer to the optimal decision tree as ${T} ^{*}$. We denote the set of internal (i.e., unfinished) nodes up to time $t$ in ${T} _{\text {A}}$ as $\mathcal {C} (t)$, and similarly as ${{\mathcal {C}}^{*}}\!(t)$ in ${T} ^{*}$.

We define $$\mathcal {C} _k = \mathcal {C} (\gamma 2^k)$$ and $${\mathcal {C} _{k}^{*}} = {{\mathcal {C}}^{*}}\!(2^{k-1})$$, for a constant $$\gamma $$ to be defined shortly. That is, we are interested in the set of unfinished nodes at the end of the $$k $$-th geometrically increasing time interval. Notice that the interval length for $$\mathcal {C} $$ is stretched by a factor of 2$$\gamma $$, compared to $${{\mathcal {C}}^{*}}$$. We define $${p} (\mathcal {C} (t)) = \sum _{S \in \mathcal {C} (t)} {p} (S)$$, i.e., the total probability of unfinished nodes at time $$t$$. Note that $${p} (\mathcal {C} (t))$$ is non-increasing as $$t$$ grows, and in the case of integral costs we have $${p} ({\mathcal {C} _{0}^{*}}) = {p} ({{\mathcal {C}}^{*}}\!(2^{-1}))=1$$, i.e., no test can be completed within a fractional cost.

The cost of some test may be truncated by the defined geometrical time intervals. To denote the actual cost of a test within an interval, we first define a path $\pi _{ik}$ in ${T} _{\text {A}}$ to be the sequence of tests involved within time $(\gamma 2^k,\infty )$ for each object $x _i$. A test $d$ selected by object $x _i$ appears in path $\pi _{ik}$ during time interval $(\gamma 2^k,\infty ) \cap (t _{i,<d},t _{i,<d}+{c} (d)]$, where $t _{i,<d}$ is the total cost before test $d$ for object $x _i$. The truncated cost of a test $d \in \pi _{ik}$ within that intersection is denoted by ${c} _{ik}(d)$. Note that ${c} _{ik}(d)\le {c} (d)$. We denote the set of tests before test $d$ in path $\pi _{ik}$ by $\pi _{ik,<d}$.

Our greedy algorithm is identical to the algorithm of Navidi et al. (2020) except that their greedy-selection score $${{Z}^{\prime }}$$ at Step 8 is replaced by the new score $$Z $$ at Step 9, in order to encourage impurity reduction of the selected tests. The key in the analysis of Navidi et al. is to show that $${p} (\mathcal {C} _{k +1}) \le 0.2 {p} (\mathcal {C} _{k}) + 3 {p} ({\mathcal {C} _{k +1}^{*}})$$, which is proven via an intermediate value $$Z _k '$$ defined below. We restate their technical result here.

#### Lemma 3

(Navidi et al. 2020, Lemma 2.4,2.5) If Algorithm 1 is executed using the greedy score ${{Z}^{\prime }}$ at Step 8, then

$$\begin{aligned} Z _k '&~\ge ~ \left( {p} (\mathcal {C} _{k +1}) - 3 {p} ({\mathcal {C} _{k +1}^{*}}) \right) {{\gamma }^{\prime }}/3, \text { and}\\ {{Z ^\infty _k}^{\prime }}&~\le ~ {p} (\mathcal {C} _{k}) {{\gamma }^{\prime }}/15, \end{aligned}$$

where ${Z _k '} = \sum _{{{\gamma }^{\prime }}2^k <t \le {{\gamma }^{\prime }}2^{k +1}} \sum _{S \in \mathcal {C} (t)} {{Z}^{\prime }}(d (S))$, ${{Z ^\infty _k}^{\prime }} = \sum _{t >{{\gamma }^{\prime }}2^k} \sum _{S \in \mathcal {C} (t)} {{Z}^{\prime }}(d (S))$, ${{\gamma }^{\prime }}=15 (1+\ln 1/\Delta +\log n)$, and $d (S)$ is the greedy test for node $S$. Besides, the first inequality holds regardless of the value of ${{\gamma }^{\prime }}$ and holds as long as $d (S)$ is a greedy test with respect to an additive score ${{Z}^{\prime }} + D$, where $D$ can be any non-negative function; the second inequality holds regardless of the choice of tests $d (S)$ in the decision tree.

Our new greedy score $$Z $$ is in an additive form required above for the first inequality. Therefore, in our case, the difficulty mainly lies in the second inequality. We can prove that a similar lemma holds for our new greedy score.

#### Lemma 4

Algorithm 1 ensures the following inequalities

$$\begin{aligned} Z _k&~\ge ~ \left( {p} (\mathcal {C} _{k +1}) - 3 {p} ({\mathcal {C} _{k +1}^{*}})\right) \gamma /3, \text { and} \\ Z ^\infty _k&~\le ~ {p} (\mathcal {C} _{k}) \gamma /15, \end{aligned}$$

where $Z _k = \sum _{\gamma 2^k <t \le \gamma 2^{k +1}} \sum _{S \in \mathcal {C} (t)} Z (d (S))$, $Z ^\infty _k = \sum _{t >\gamma 2^k} \sum _{S \in \mathcal {C} (t)} Z (d (S))$, $\gamma =15 (1+\ln 1/\Delta +\log n +\lambda \epsilon _h )$, and $d (S)$ is the greedy test for node $S$.

#### Proof

The first inequality is easy to show. Notice that compared to $$Z '$$, $$Z $$ introduces a third term of impurity reduction $$D$$ in Eq. (2), which is always non-negative (see Sect. 3) and thus $$Z (d) \ge {{Z}^{\prime }}(d)$$. Thus, the first inequality in Lemma 3 also holds for the tree generated by the new greedy score. Since the first inequality in Lemma 3 does not depend on the value of $${{\gamma }^{\prime }}$$, we replace it with the new $$\gamma $$, which completes the proof.

The second inequality requires more work. We denote the sum of the $$D $$ terms in $$Z ^\infty _k $$ by

$$ G = \lambda \sum _{t >\gamma 2^k} \sum _{S \in \mathcal {C} (t)} \frac{{p} (S)}{{c} (d (S))} \left( h (S) - h (S \mid d (S))\right) . $$

From Lemma 3 we know that $Z ^\infty _k- G \le {{\gamma }^{\prime }} {p} (\mathcal {C} _{k}) /15$.

We now upper bound the additional term *G*. We omit $S$ in $d (S)$ when it is clear from the context.

3 $$\begin{aligned} G&= \lambda \sum _{t>\gamma 2^k} \sum _{S \in \mathcal {C} (t)} \frac{{p} (S)}{{c} (d)} \left( h (S) - h (S \mid d)\right) \nonumber \\&= \lambda \sum _{t>\gamma 2^k} \sum _{S \in \mathcal {C} (t)} \frac{{p} (S)}{{c} (d)} \left( h (S) - \sum _{v \in [\nu _{d}]} \frac{{p} (S _{d}^v)}{{p} (S)} h (S _{d}^v)\right) \nonumber \\&= \lambda \sum _{t>\gamma 2^k} \sum _{S \in \mathcal {C} (t)} \frac{{p} (S)}{{c} (d)} \left( \frac{1}{{p} (S)} \sum _{x \in S} {p} (x) h _{\ell (x)}(S)\right. \nonumber \\&\qquad \left. - \sum _{v \in [\nu _{d}]} \frac{{p} (S _{d}^v)}{{p} (S)} \frac{1}{{p} (S _{d}^v)} \sum _{x \in S _{d}^v} {p} (x) h _{\ell (x)}(S _{d}^v) \right) \nonumber \\&= \lambda \sum _{t>\gamma 2^k} \sum _{S \in \mathcal {C} (t)} \frac{1}{{c} (d)} \left( \sum _{x \in S} {p} (x) h _{\ell (x)}(S) - \sum _{v \in [\nu _{d}]} \sum _{x \in S _{d}^v} {p} (x) h _{\ell (x)}(S _{d}^v) \right) \nonumber \\&= \lambda \sum _{t >\gamma 2^k} \sum _{S \in \mathcal {C} (t)} \sum _{x \in S} \frac{{p} (x)}{{c} (d)} \left( h _{\ell (x)}(S) - h _{\ell (x)}\!\left( S _{d}^{d (x)}\right) \right) \nonumber \\&= \lambda \sum _{S \in \mathcal {C} _k} \sum _{i:x _i\in S} {p} (x _i) \sum _{d \in \pi _{ik}} \frac{{c} _{ik}(d)}{{c} (d)} \left( h _{\ell (x _i)}\!\left( S ^{(i)}_{\pi _{ik,<d}}\right) - h _{\ell (x _i)}\!\left( S ^{(i)}_{\pi _{ik,<d}\cup \{d \}}\right) \right) \end{aligned}$$

4 $$\begin{aligned}&\le \lambda \sum _{S \in \mathcal {C} _k} \sum _{i:x _i\in S} {p} (x _i) \sum _{d \in \pi _{ik}} \left( h _{\ell (x _i)}\!\left( S ^{(i)}_{\pi _{ik,<d}}\right) - h _{\ell (x _i)}\!\left( S ^{(i)}_{\pi _{ik,<d}\cup \{d \}}\right) \right) \end{aligned}$$

5 $$\begin{aligned}&\le \lambda \sum _{S \in \mathcal {C} _k} \sum _{i:x _i\in S} {p} (x _i)\, h _{\ell (x _i)}(S) \\&\le \lambda \sum _{S \in \mathcal {C} _k} \sum _{i:x _i\in S} {p} (x _i)\, \epsilon _h \nonumber \\&= \lambda \, {p} (\mathcal {C} _k)\, \epsilon _h ,\nonumber \end{aligned}$$

where step (3) follows by enumerating the summands in a different order, step (4) is due to ${c} _{ik}(d) \le {c} (d)$, and step (5) follows by considering the telescoping series of the impurity reduction along a path of an object. Putting everything together gives

$$\begin{aligned} Z ^\infty _k = G + (Z ^\infty _k-G) \le \lambda {p} (\mathcal {C} _{k}) \epsilon _h + {p} (\mathcal {C} _{k}) {{\gamma }^{\prime }}/15 = {p} (\mathcal {C} _{k}) \gamma /15. \end{aligned}$$

$$\square $$

Next, we use another simple lemma that provides an upper bound on the expected cost $C_{\text {A}}$ of ${T} _{\text {A}}$, and a lower bound on the optimal cost $C^{*}$ of ${T} ^{*}$. This result is a consequence of the geometrical division of time. For example, to obtain an upper bound for $C_{\text {A}}$, we assume that the set of unfinished nodes stays the same as $\mathcal {C} (\gamma 2^{k})$ during the time interval $(\gamma 2^k,\gamma 2^{k +1}]$. Recall that ${p} (\mathcal {C} (t))$ is a non-increasing function of time $t$.

#### Lemma 5

(Navidi et al. 2020, Lemma 2.2) The expected cost $C_{\text {A}}$ of the tree ${T} _{\text {A}}$ produced by Algorithm 1, and the cost $C^{*}$ of the optimal tree ${T} ^{*}$ for the $\hbox {NGI}$ problem, satisfy the following inequalities

$$\begin{aligned} C_{\text {A}}&~\le ~ \gamma \sum _{k \ge 0} 2^k {p} (\mathcal {C} _k) + \gamma , \text { and} \\ C^{*}&~\ge ~ \frac{1}{2} \sum _{k \ge 0} 2^{k-1} {p} ({\mathcal {C} _{k}^{*}}). \end{aligned}$$

We are now ready to prove our main result, Theorem 1, stated in Sect. 5. The proof relies on combining the results of Lemma 4 with the upper and lower bounds provided by Lemma 5.

#### Proof

(Theorem 1)   From Lemma 4, we obtain

$$ \left( {p} (\mathcal {C} _{k +1}) - 3 {p} ({\mathcal {C} _{k +1}^{*}})\right) \gamma /3 ~\le ~ Z _k ~\le ~ Z ^\infty _k ~\le ~ {p} (\mathcal {C} _{k}) \gamma /15. $$

By rearranging terms, we get

$$ {p} (\mathcal {C} _{k +1}) ~\le ~ 0.2\, {p} (\mathcal {C} _{k}) + 3\, {p} ({\mathcal {C} _{k +1}^{*}}). $$

Define $Q=\gamma \sum _{k \ge 0} 2^k {p} (\mathcal {C} _k) + \gamma$, i.e., the upper bound of $C_{\text {A}}$. We have

$$\begin{aligned} Q&= \gamma \sum _{k \ge 1} 2^k {p} (\mathcal {C} _k) + \gamma \left( {p} (\mathcal {C} _0)+1\right) \\&\le \gamma \sum _{k \ge 1} 2^k \left( 0.2\, {p} (\mathcal {C} _{k-1}) + 3\, {p} ({\mathcal {C} _{k}^{*}})\right) + \gamma \left( {p} (\mathcal {C} _0)+1\right) \\&\le \gamma \sum _{k \ge 0} 2^{k}\, 0.4\, {p} (\mathcal {C} _{k}) + \gamma \frac{1}{2}\, \sum _{k \ge 1} 2^{k-1}\, 12\,{p} ({\mathcal {C} _{k}^{*}}) + \gamma \left( {p} (\mathcal {C} _0)+1\right) \\&= \gamma \sum _{k \ge 0} 2^{k}\, 0.4\, {p} (\mathcal {C} _{k}) + \gamma \frac{1}{2} \sum _{k \ge 0} 2^{k-1}\, 12\,{p} ({\mathcal {C} _{k}^{*}}) - 3\,\gamma \,{p} ({\mathcal {C} _{0}^{*}}) + \gamma \left( {p} (\mathcal {C} _0)+1\right) \\&\le \gamma \sum _{k \ge 0} 2^{k}\, 0.4 {p} (\mathcal {C} _{k}) + \gamma \frac{1}{2}\, \sum _{k \ge 0} 2^{k-1}\, 12\,{p} ({\mathcal {C} _{k}^{*}}) \\&\le 0.4\, Q + 12\,\gamma \, C^{*}, \end{aligned}$$

where we note that ${p} ({{\mathcal {C} _{0}}^{*}})=1$ and ${p} (\mathcal {C} _0)\le 1$. Together with Lemma 5, we obtain

$$ C_{\text {A}} ~\le ~ Q ~\le ~ \frac{12}{0.6} \, \gamma \, C^{*} ~=~ 20\, \gamma \, C^{*}. $$

$$\square $$

## Experimental evaluation

In this section, we evaluate the performance of our enhanced decision-tree algorithms by comparing them against strong baselines on a large collection of real-world datasets. Some additional experimental results are presented in the Appendix, including further experimental results for $\hbox {CART}$ (Section C), further experimental results on tree size (Section D), additional statistical tests (Section E), and more visual examples (Section G). Our implementation and pre-processing scripts can be found in a Github repository.^2^

**Datasets** We evaluate our methods on 20 datasets from the UCI Machine Learning Repository (Dua and Graff 2017) and OpenML (Vanschoren et al. 2013). Information about the datasets is shown in Table 1. We experiment with datasets containing up to 0.6 million objects and 5 thousand features. We set the limit $\theta$ to be 0.005 for all datasets except for small ones, whose $\theta$ are set accordingly so that the minimum leaf size is 2. For all datasets, 70% of the data points are used for training, 10% for validation and the rest for testing. Numerical features are categorized into multiple bins by the *k*-means strategy, which can adapt to uneven data distributions. All categorical features are then binarized to avoid biases towards features with a large number of levels (Strobl et al. 2007). All identical objects are coalesced into a single object, and the sampling probability is set accordingly. To fulfill the realizability assumption, the majority class is assigned to each identical data point in the training set, which may have different classes otherwise, due to noise or feature discretization. Apart from the original datasets with unit test cost, we additionally create more challenging scenarios, where each test cost is independently drawn from the set $\{1,\ldots ,10\}$.

**Table 1:** Datasets statistics; $$n, m, k $$: number of data points, binary features and classes

Dataset	*n*	$m$	$k$
iris	150	20	3
ilpd	583	46	2
breast-w	699	45	2
tic-tac-toe	958	27	2
obesity	2111	58	7
bioresponse	3751	5333	2
spambase	4601	285	2
phoneme	5404	25	2
musk	6598	830	2
speed-dating	8378	733	2
phishing-websites	11,055	46	2
shoppers	12,330	454	2
letter	20,000	80	26
default	30,000	112	2
bank-marketing	45,211	76	2
electricity	45,312	42	2
firewall	65,532	55	4
dota2	92,649	394	2
diabetic	101,766	264	3
covertype	581,012	94	7

Numerical features are categorized into 5 bins by the *k*-means strategy

**Algorithms and baselines** A summary of the algorithms is displayed in Table 2. The algorithms that implement the proposed approach are denoted as *enhanced* *C*4.5 ($\hbox {EC}4.5$) and *enhanced* *CART* ($\hbox {ECART}$). Baselines include the following:

- The $\hbox {ASR}$ method (Navidi et al. 2020), which is the greedy algorithm without the newly-introduced impurity-reduction term.
- Impure Pairs ($\hbox {IP}$), which maximizes the reduction in the number of impure pairs at each split, i.e., the unweighted edge cut among different classes (Golovin et al. 2010; Cicalese et al. 2014).
- $\hbox {BAL}$, which is an unsupervised balanced-tree algorithm that greedily selects the test that splits the current node most evenly.
- The two traditional algorithms $\hbox {C}4.5$ and $\hbox {CART}$, and their cost-benefit versions that select a test using a cost-weighted criterion (denoted with a prefix ‘C’).

**Table 2:** Summary of competing algorithms

Algorithm	Brief description
$\hbox {ASR}$	Greedy without impurity reduction (Navidi et al. 2020)
$\hbox {IP}$	Greedy in reducing the number of impure pairs (Golovin et al. 2010)
$\hbox {BAL}$	Greedy in the most balanced split
[p][C]$\hbox {CART}$	Traditional $\hbox {CART}$ (Breiman et al. 1984)
[p][C]$\hbox {C}4.5$	Traditional $\hbox {C}4.5$ (Quinlan 1993)
[p]$\hbox {ECART}$	Enhanced $\hbox {CART}$ (proposed method)
[p]$\hbox {EC}4.5$	Enhanced $\hbox {C}4.5$ (proposed method)

Prefix ‘p-’ indicates a variant with post-pruning

To ensure a meaningful comparison, we measure performance for all methods based on the same stopping criteria. All algorithms perform two-way splitting. Splitting of tree nodes stops if homogeneity is achieved or if the minimum-probability limit is reached. We examine the performance of $\hbox {C}4.5$ and $\hbox {CART}$ with post-pruning (denoted with a prefix ‘p’) or without. We adopt the standard *minimal cost-complexity pruning* approach (Breiman et al. 1984), which prunes a tree node having many leaves if its impurity is no much larger than the total impurity of its leaves. The parameter that controls the stringency of the pruning is determined by cross-validation over a logspace from $10^{-5}$ to 1.

**Fig. 2:**
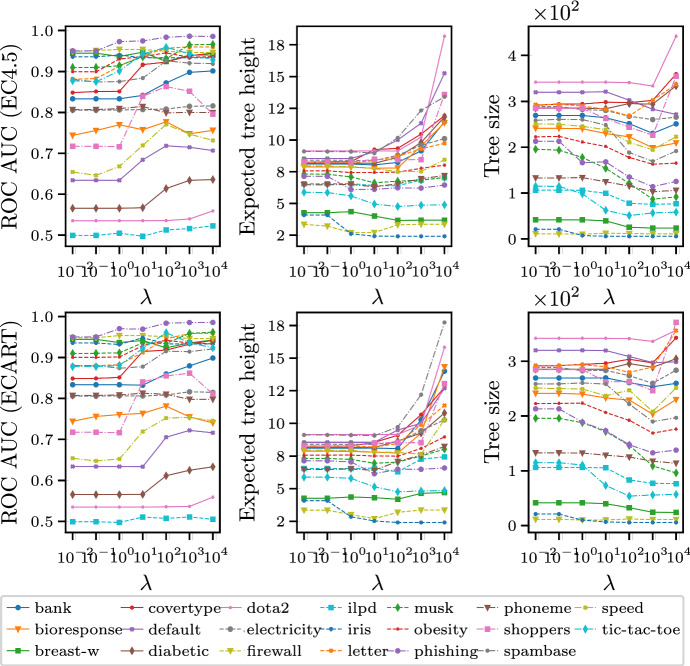
The effect of the trade-off hyperparameter $\lambda$. ROC AUC is on validation data, and expected tree height or tree size on training data

The only hyperparameter in our algorithm ($\lambda$) controls the trade-off between complexity and discrimination. The effect of $\lambda$ is summarized in Fig. 2. For large values of $\lambda$ our algorithms turn into the traditional tree-induction algorithms $\hbox {C}4.5$ and $\hbox {CART}$; on the other hand, if $\lambda$ is zero, our algorithms turn into the greedy algorithm for the $\hbox {ASR}$ problem. As we are working with a bi-criteria optimization problem, there is no golden rule in deciding the best value of the hyperparameter. In this experiment, we aim to decide a value of $\lambda$ that preserves comparable accuracy while reduces the complexity as much as possible. Thus, we tune the hyperparameter $\lambda$ by starting with a large $\lambda$ and gradually decreasing it before a significant drop (larger than 1%) is seen in the predictive accuracy over the validation set. Note also that $\lambda$ is invariant to the data size, as the greedy score only depends on the distributions before and after the split.

**Fig. 3:**
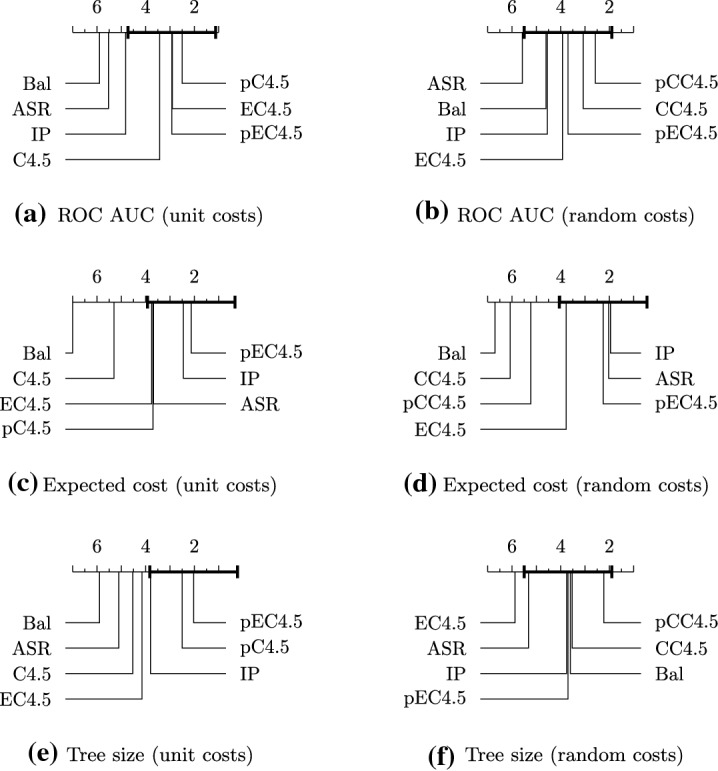
Critical difference for the Bonferroni-Dunn test on significance level $\alpha =0.05$ for average ranks of algorithms among 20 tested datasets. Methods closer to the right end have a better rank. The method that is compared with other methods is p$\hbox {EC}4.5$, and methods lying outside the thick interval are significantly different from p$\hbox {EC}4.5$

**Fig. 4:**
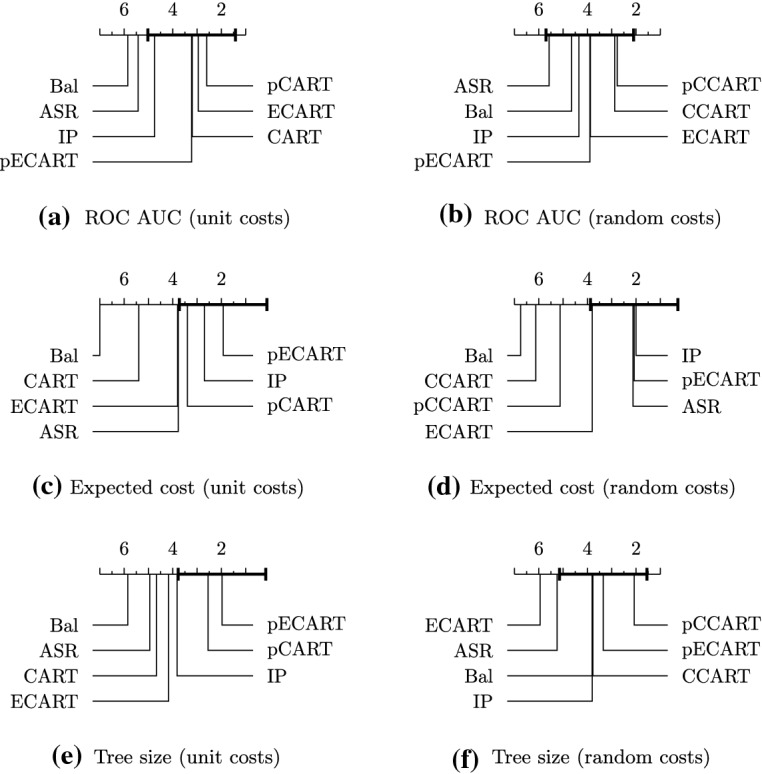
Critical difference for the Bonferroni-Dunn test on significance level $\alpha =0.05$ for average ranks of algorithms among 20 tested datasets. Methods closer to the right end have a better rank. The method that is compared with other methods is p$\hbox {ECART}$, and methods lying outside the thick interval are significantly different from p$\hbox {ECART}$

**Fig. 5:**
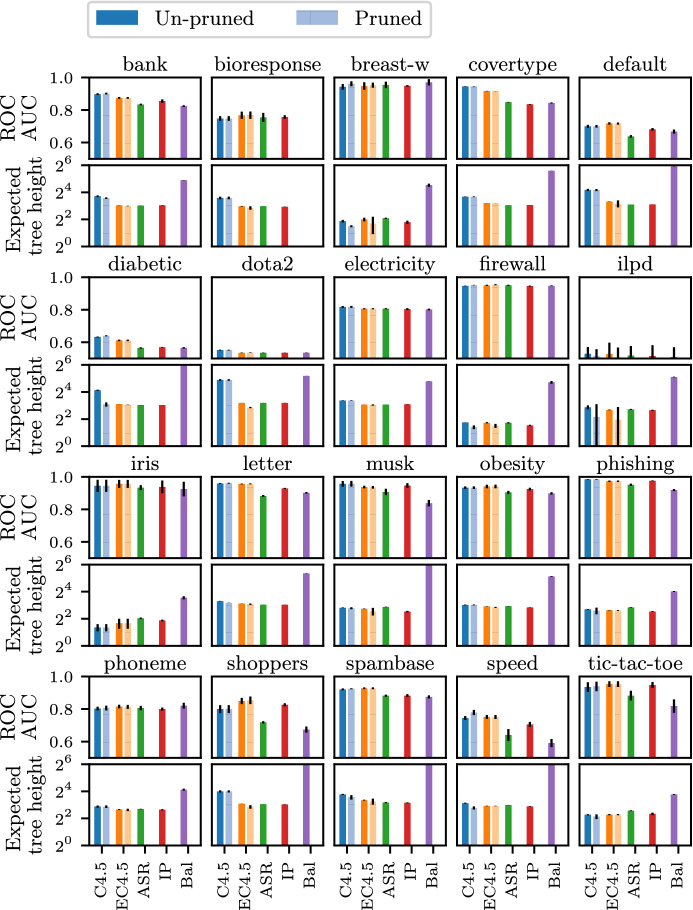
Performance results with unit test costs. All plots in the same row share the same x- and y-axes. Error bars are also shown

**Results** We evaluate all methods using $\hbox {ROC AUC}$ as a measure of predictive power, expected cost as a measure of tree complexity, and tree size (i.e., the number of tree nodes) as an auxiliary measure of global tree complexity. A full result on tree size is deferred to Section D in Appendix. Reported results, shown in Fig. 5, are averages over 5 executions with random train-test splits. We conduct the Bonferroni-Dunn test with significance level $\alpha =0.05$ for average ranks (Demšar 2006), and report the *critical difference diagram* in Figs. 3 and 4, where the proposed method p$\hbox {EC}4.5$ (or p$\hbox {ECART}$) is tested against the other methods, and methods closer to the right end have a better rank. We see that the predictive power and tree complexity of p$\hbox {ECART}$ and p$\hbox {EC}4.5$ are statistically not significantly different from the respective best performer, while it is significantly better than most other baselines. Two methods $\hbox {C}4.5$ and $\hbox {CART}$ lead to similar behavior; we focus on $\hbox {C}4.5$ below and discuss its results in details. Full results for $\hbox {CART}$ and its enhancements are presented in the Appendix, Section C.

It can be seen that post-pruning has a noticeable positive effect on both the accuracy and complexity for the $\hbox {C}4.5$ algorithm. However, even after post-pruning, p$\hbox {C}4.5$ is still ranked closely to un-pruned $\hbox {EC}4.5$ in terms of the expected cost, and in some datasets, the expected cost of p$\hbox {C}4.5$ is about two times larger than that of $\hbox {EC}4.5$ in Fig. 5. This is reasonable because post-pruning mainly removes tree nodes near the bottom, but fails to rescue early bad splits near the root. On the other hand, post-pruning is significantly more beneficial than impurity reduction for the global tree size. Also note that post-pruning has less effect on $\hbox {EC}4.5$ in terms of accuracy, which indicates that un-pruned $\hbox {EC}4.5$ alone is robust to overfitting.

The decision tree produced by $\hbox {BAL}$ is the worst in both aspects. This is expected for predictive power as $\hbox {BAL}$ is an unsupervised method, but it is quite surprising for complexity. It turns out that $\hbox {BAL}$ often has to keep expanding a balanced tree until the minimum leaf size is reached, as tree nodes rarely achieve homogeneity. This behavior reinforces the argument that discriminative tests help accelerating termination and reducing expected cost.

The $\hbox {IP}$ algorithm achieves better performance in both aspects than the $\hbox {ASR}$ algorithm. However, $\hbox {IP}$ has a too strong bias towards a balanced split, that it favors a random test over a discriminative one in the example we provide in Section A. This bias is also reflected in Fig. 5 where it falls behind $\hbox {ECART}$ by more than 10% accuracy in some datasets. By further statistical tests we conduct in Section E, the predictive power of $\hbox {ASR}$ and $\hbox {IP}$ are statistically indistinguishable from the unsupervised $\hbox {BAL}$.

Finally, regarding running time, algorithm $\hbox {EC}4.5$ typically runs 3–4 times longer than $\hbox {C}4.5$, but there are instances that the latter algorithm constructs very skewed trees and it takes more time to complete (details in Appendix, Section F).

**Fig. 6:**
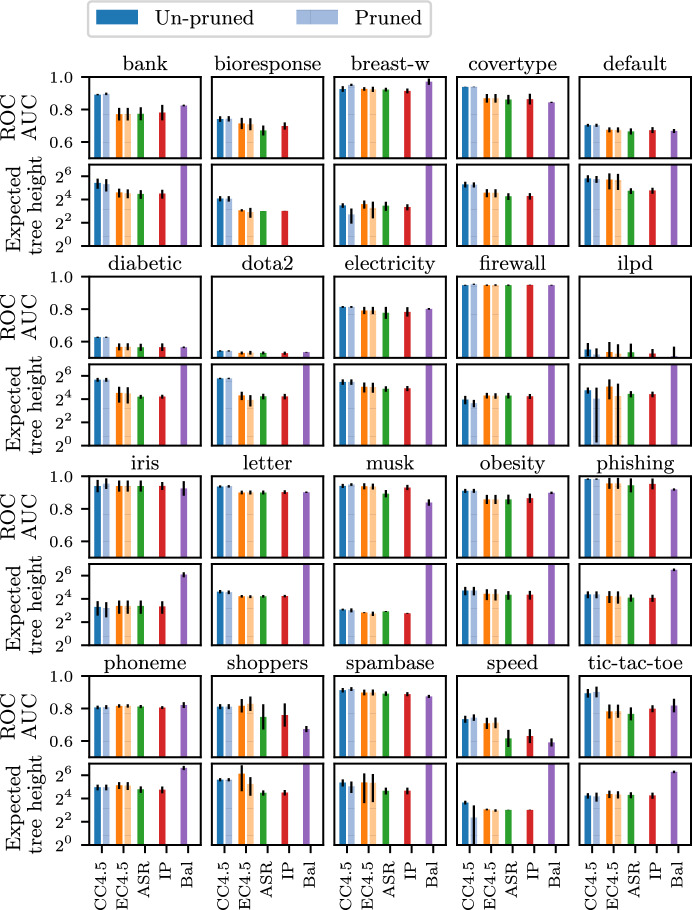
Performance results with non-uniform test costs

The benefit of the proposed method becomes more pronounced in non-uniform-cost scenarios, shown in Fig. 6. It turns out that the cost-benefit traditional trees fail to reduce the expected cost, which indicates the need for more sophisticated techniques like ours to tackle non-uniform costs. Our algorithms obtain comparable predictive power, while achieving up to 90% lower expected cost than the traditional trees.

We conclude that our enhancement, given in the form of a regularizer, strikes an excellent balance between predictive power and expected tree height.

## Conclusion

In this paper, we proposed a novel algorithm to construct a general decision tree with asymptotically tight approximation guarantee on expected cost under mild assumptions. The algorithm can be used to assimilate many existing standard impurity functions so as to enhance their corresponding splitting criteria with a complexity guarantee. Through empirical evaluation on various datasets and scenarios, we verified the effectiveness of our algorithm both in terms of accuracy and complexity. Potential future directions include the study of different complexity measures, further termination criteria, and incorporating a broader family of impurity functions.

## Figures and Tables

**Fig. 7:**
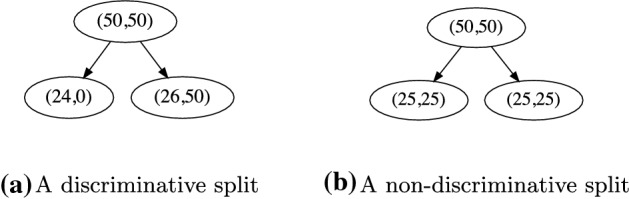
A simple example demonstrating that $\hbox {ASR}$ split criteria may lead to non-discriminative decision trees

**Fig. 8:**
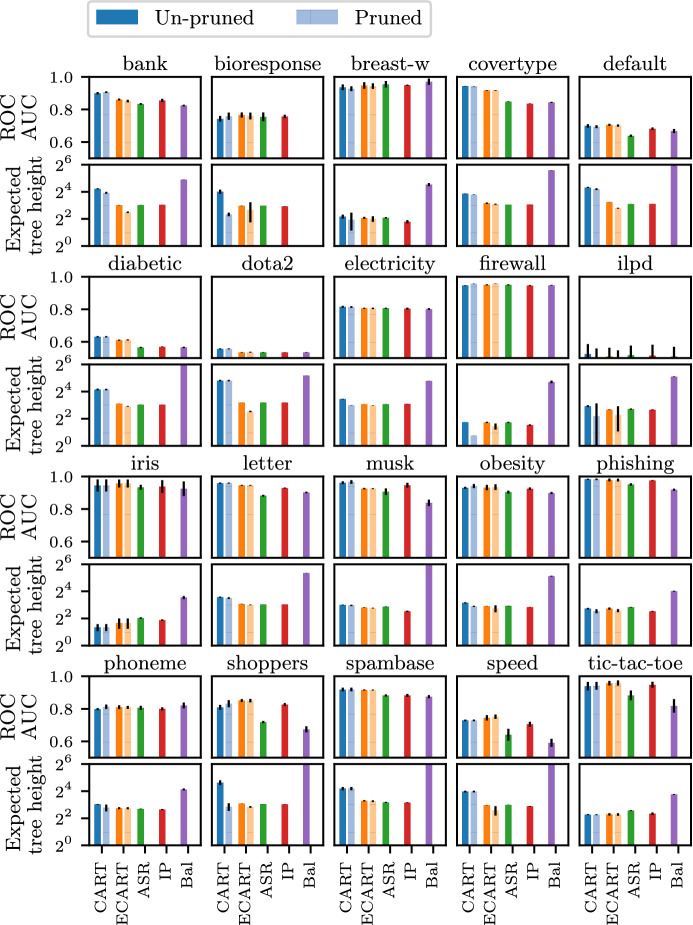
Performance results with unit test costs. All plots in the same row share the same x- and y-axes. Error bars are also shown

**Fig. 9:**
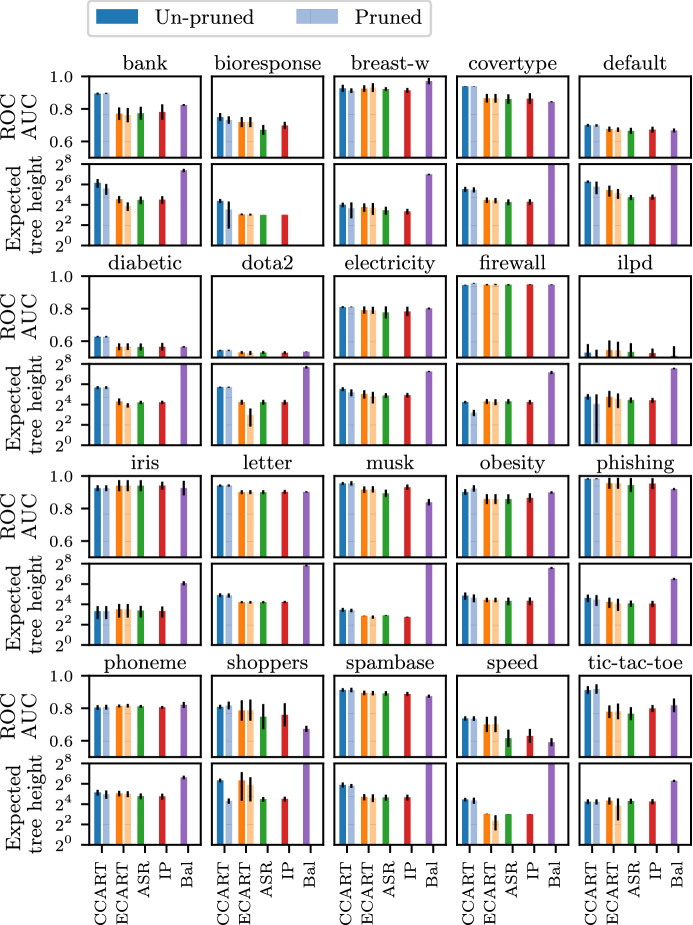
Performance results with non-uniform test costs

**Fig. 10:**
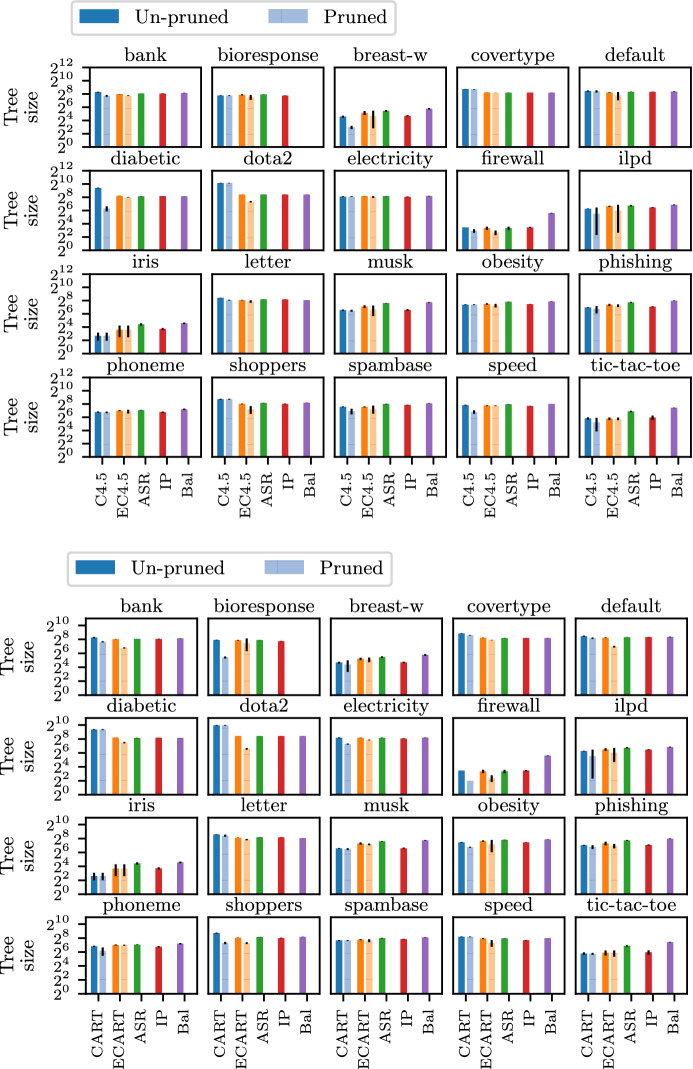
Performance results with unit test costs. All plots in the same row share the same x- and y-axes. Error bars are also shown

**Fig. 11:**
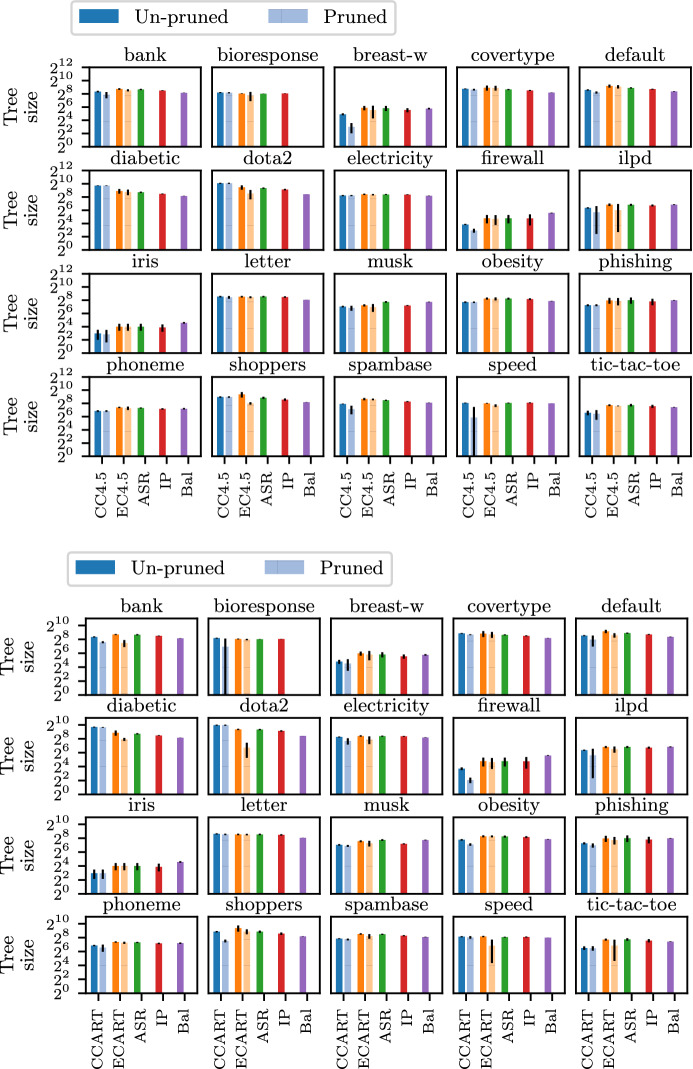
Performance results with non-uniform test costs

**Fig. 12:**
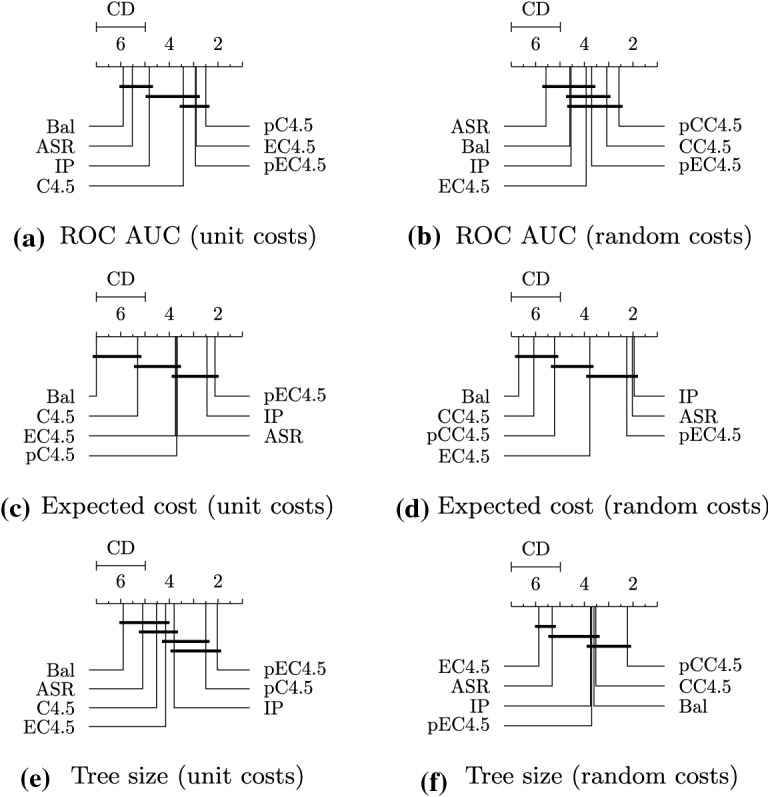
Critical difference for the Nemenyi test on significance level $\alpha =0.05$ for average ranks of algorithms among 20 tested datasets. Methods closer to the right end have a better rank. Any pair of methods which are not connected with an horizontal line have an average rank that is different with statistical significance

**Fig. 13:**
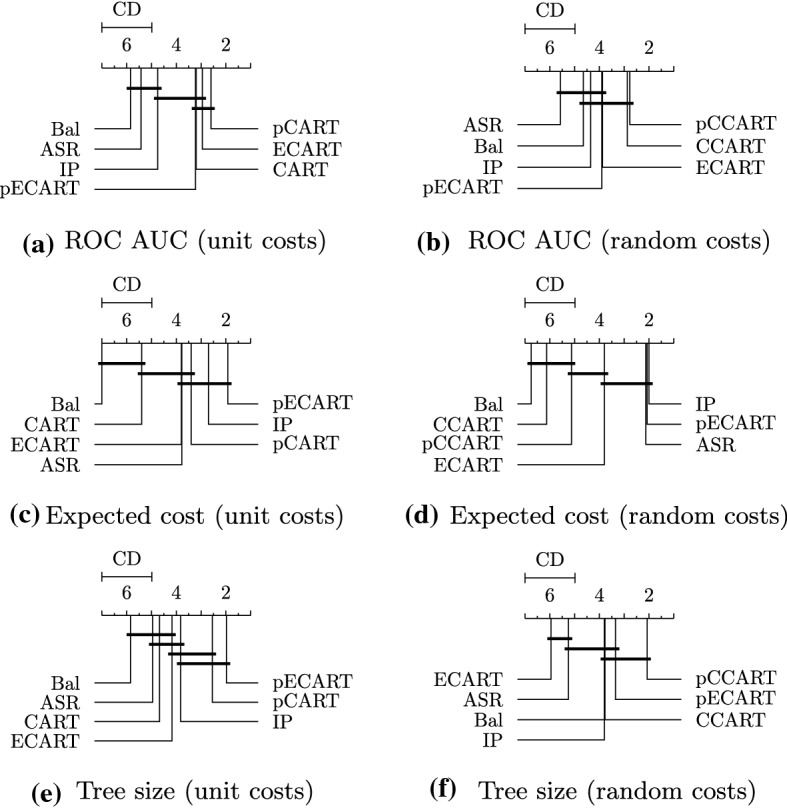
Critical difference for the Nemenyi test on significance level $\alpha =0.05$ for average ranks of algorithms among 20 tested datasets. Methods closer to the right end have a better rank. Any pair of methods which are not connected with an horizontal line have an average rank that is different with statistical significance

**Fig. 14:**
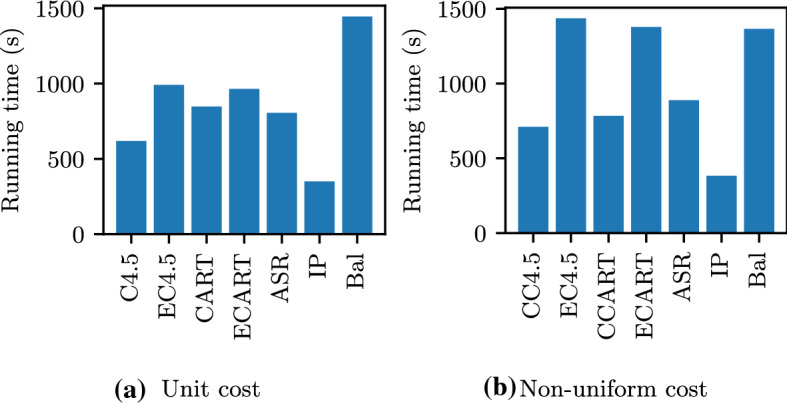
Running time, average over all datasets

**Fig. 15:**
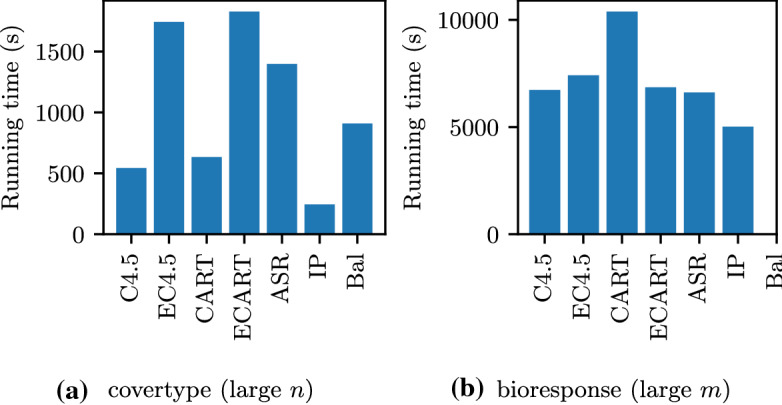
Running time on selected datasets

**Fig. 16:**
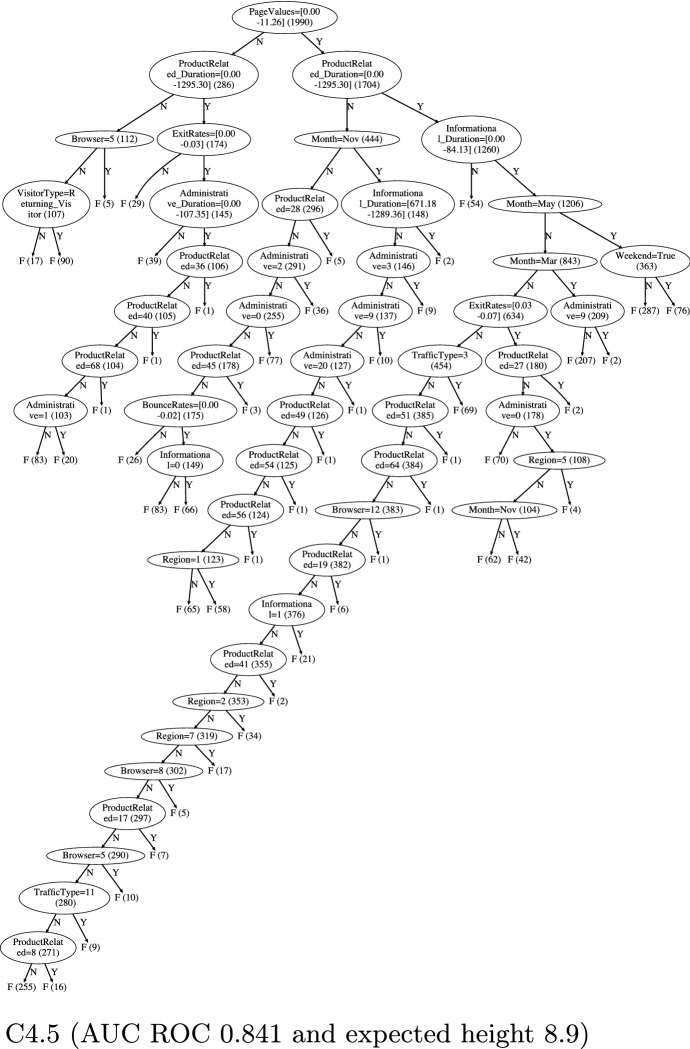
Visualization of $\hbox {EC}4.5$ decision tree for shoppers dataset

**Fig. 17:**
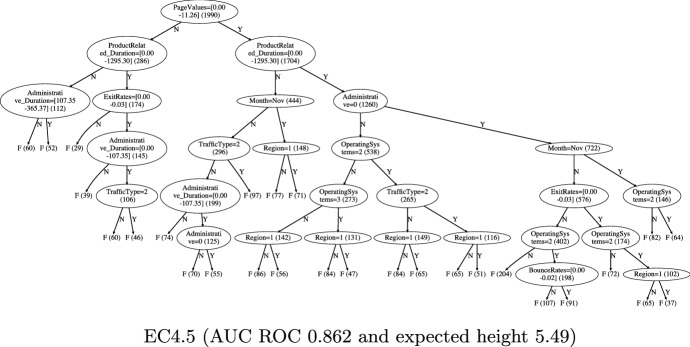
Visualization of $\hbox {EC}4.5$ decision tree for shoppers dataset

**Fig. 18:**
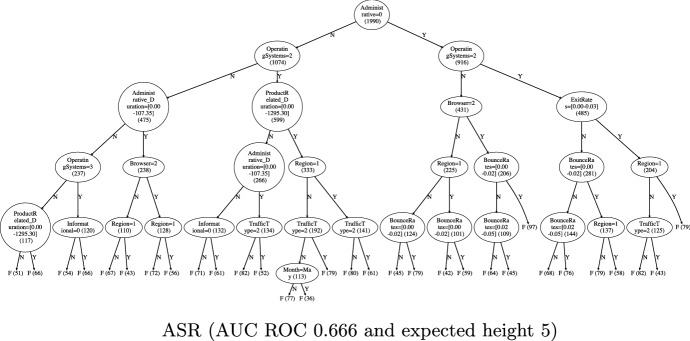
Visualization of $\hbox {ASR}$ decision tree for shoppers dataset

**Fig. 19:**
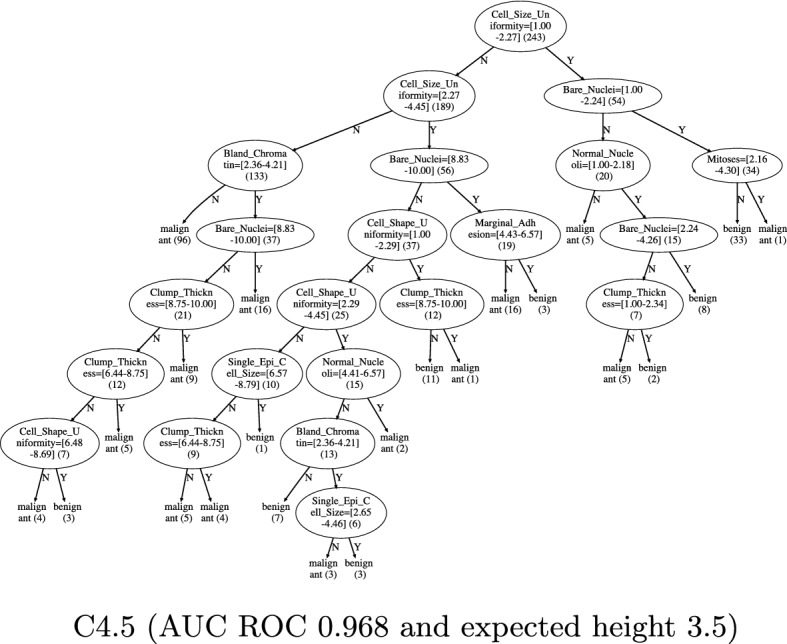
Visualization of $\hbox {C}4.5$ decision tree for breast-w dataset

**Fig. 20:**
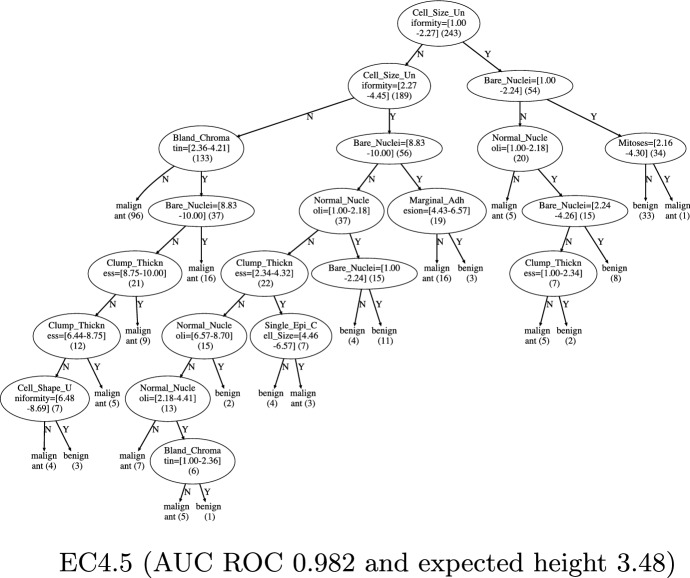
Visualization of $\hbox {EC}4.5$ decision tree for breast-w dataset

**Fig. 21:**
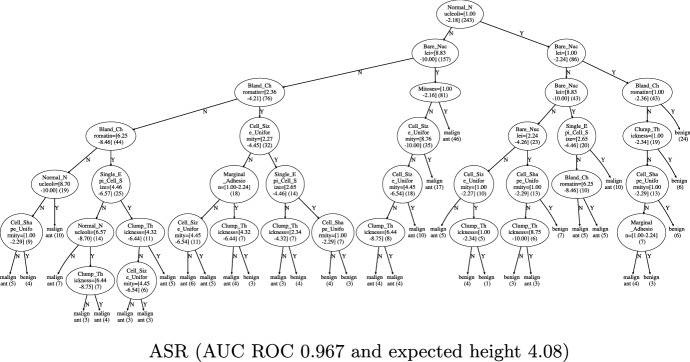
Visualization of $\hbox {ASR}$ decision tree for breast-w dataset

**Fig. 22:**
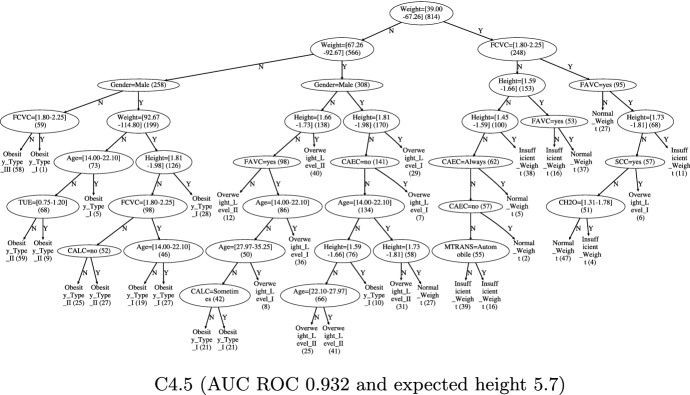
Visualization of $\hbox {C}4.5$ decision tree for obesity dataset

**Fig. 23:**
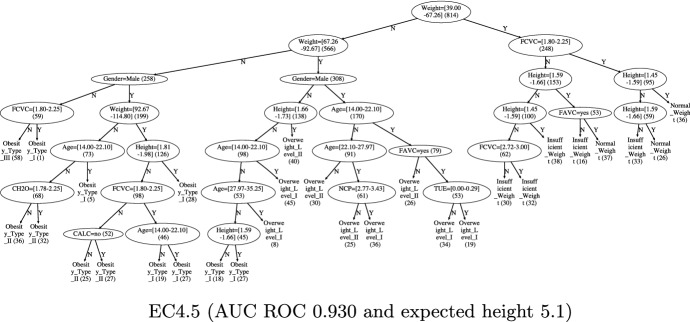
Visualization of $\hbox {EC}4.5$ decision tree for obesity dataset

**Fig. 24:**
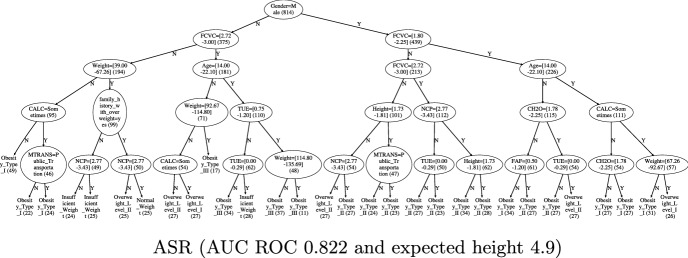
Visualization of $\hbox {ASR}$ decision tree for obesity dataset

**Fig. 25:**
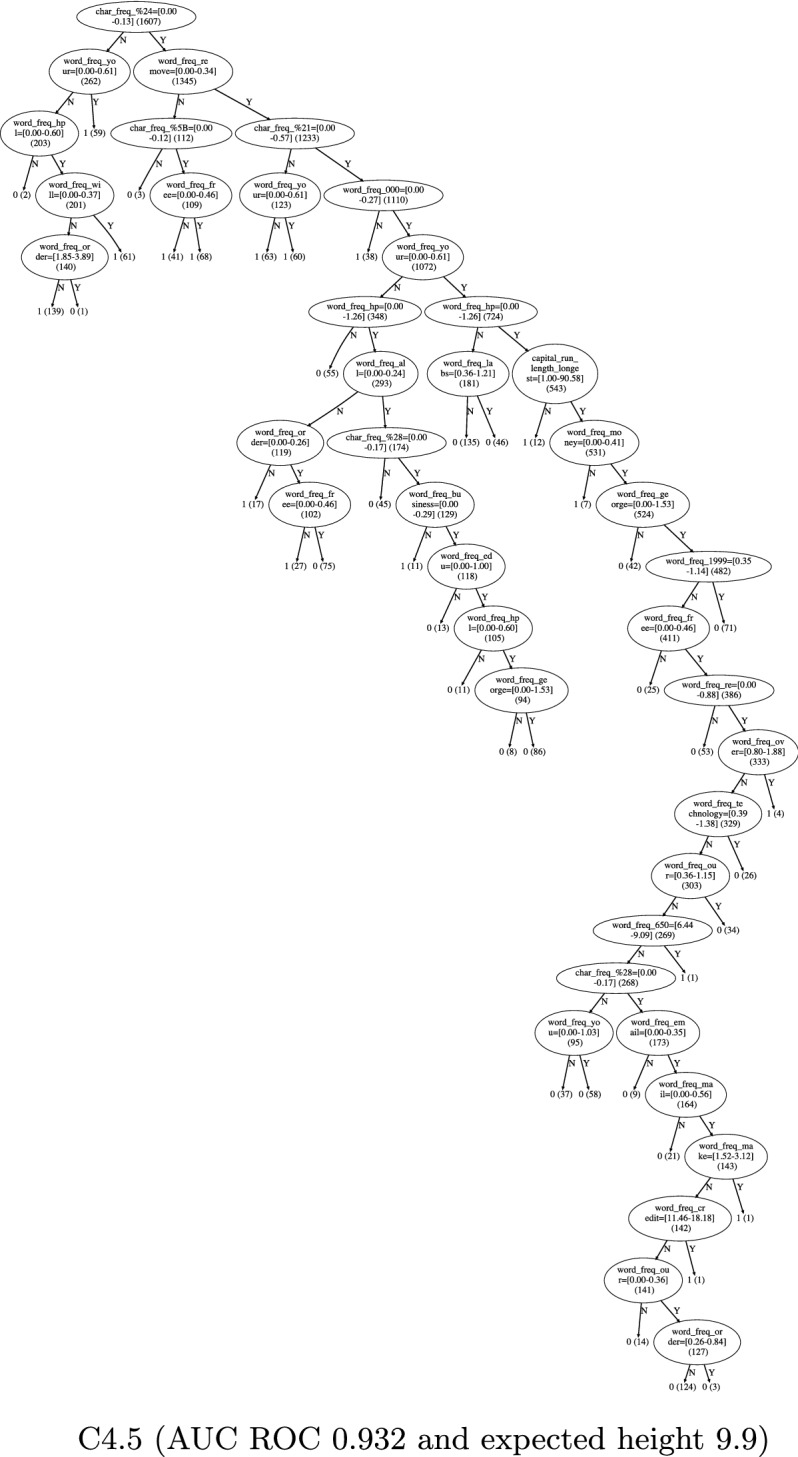
Visualization of $\hbox {C}4.5$ decision tree for spambase dataset

**Fig. 26:**
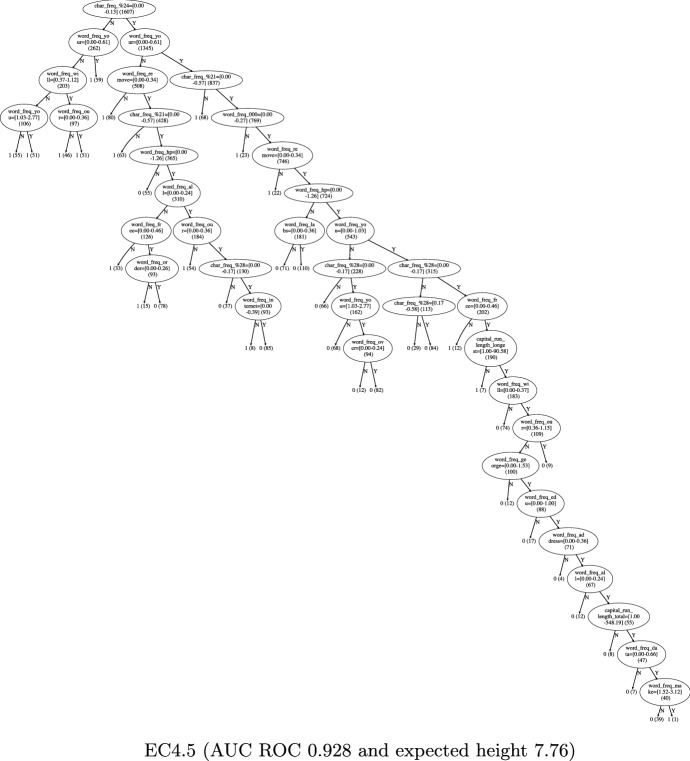
Visualization of $\hbox {EC}4.5$ decision tree spambase dataset

**Fig. 27:**
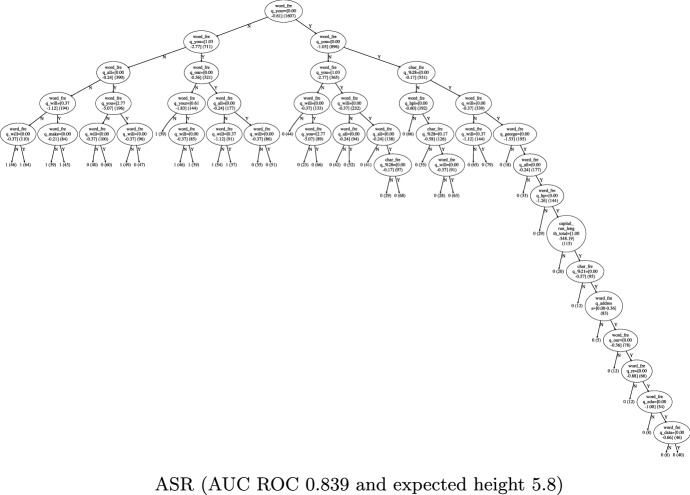
Visualization of $\hbox {ASR}$ decision tree for spambase dataset

**Fig. 28:**
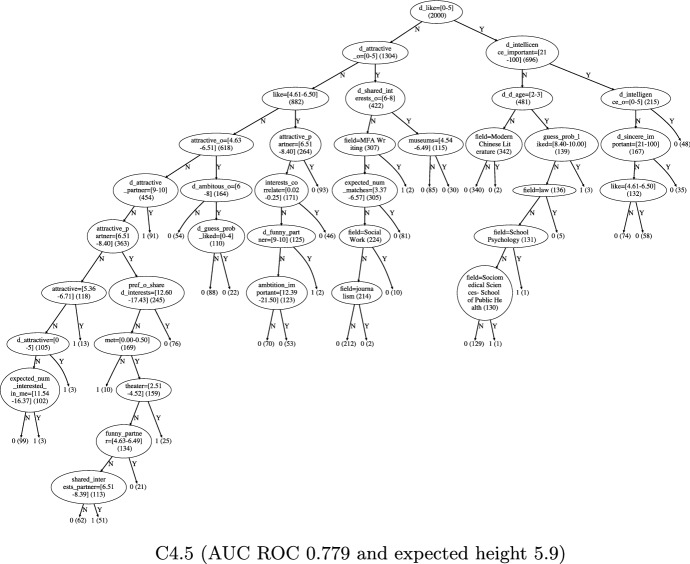
Visualization of $\hbox {C}4.5$ decision tree for speed-dating dataset

**Fig. 29:**
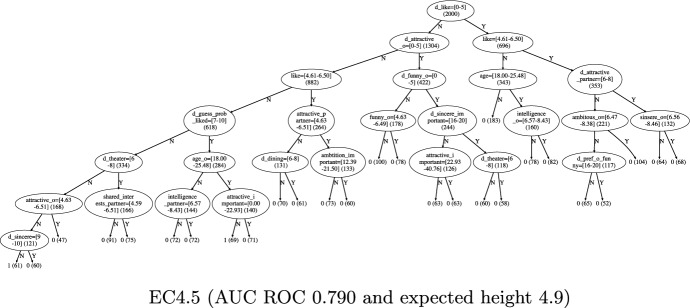
Visualization of $\hbox {EC}4.5$ decision tree for speed-dating dataset

**Fig. 30:**
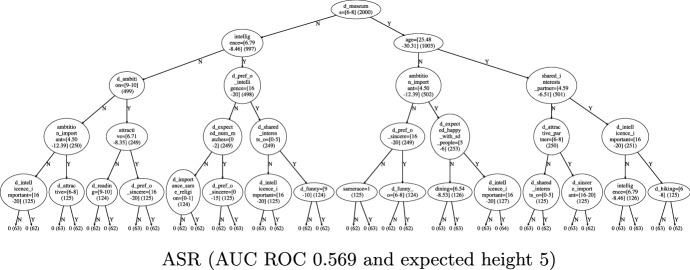
Visualization of $\hbox {ASR}$ decision tree for speed-dating dataset

## Data Availability

All datasets we use are publicly available in the UCI Machine Learning Repository (Dua and Graff 2017) and OpenML (Vanschoren et al. 2013).

## Appendix A Split criteria for adaptive submodular ranking (ASR) method may lead to non-discriminative decision trees

We present a simple example demonstrating that the $\hbox {ASR}$ method, used for the group identification problem where the aim is to minimize the expected cost of a decision tree, may not select discriminative splits, which in turn may lead to decision trees with low predictive power. The splitting criterion of $\hbox {ASR}$ consists of two terms (see Sect. 4 for more details). One term encourages balanced partitions and does not use label information. For the other term, two criteria have been considered in the literature: (1) maximize reduction in the number of *heterogeneous pairs* of objects after a split, or (2) maximize the number of excluded objects in other classes. These two criteria turn out to be equivalent up to a constant factor of 2 by a simple double counting. Apparently, when the number of heterogeneous pairs drops to zero or each object separates from all objects in other classes, we obtain perfect accuracy (in training data). Note that even random splits can lead to perfect accuracy as long as the tree is fully expanded. Here we discuss the second term, as random splits actually optimize balance, on expectation.

Our example is shown in Fig. 7, where we demonstrate two possible splits on a node. As we will see, $\hbox {ASR}$ favors the non-discriminative split Fig. 7b over the more discriminative split Fig. 7a. We assume two classes, and we write (*x*, *y*) to indicate the number of objects from the two different classes in a tree node. We now discuss the first criterion. In the left tree Fig. 7a, the root, the left, and the right node have $50\times 50$, 0, and $26\times 50$ heterogeneous pairs, respectively. In the right tree Fig. 7b, there are $50\times 50$, $25\times 25$, and $25\times 25$ pairs, respectively. The left split decreases the number of heterogeneous pairs by $50\times 50-0-26\times 50=24\times 50$. The right split decreases the number of heterogeneous pairs by $50\times 50-25\times 25-25\times 25=50\times 25$. Thus, the $\hbox {ASR}$ criterion will select the (non-discriminative) split Fig. 7b.

## Appendix B Impurity reduction is non-negative

By the concavity property of $h$, it is easy to show that the impurity-reduction function $d (S,d)$ is non-negative, for any tree node $S$ and test $d$. In particular, we have

B1 $$\begin{aligned} d (S,d)&~=~ h (S) - h (S \mid d) \nonumber \\&~=~ h (S) - \sum _{v \in [\nu _d ]} \frac{{p} (S _d ^v)}{{p} (S)} h (S _d ^v) \nonumber \\&~=~ h ({p} _S) - \sum _{v \in [\nu _d ]} \frac{{p} (S _d ^v)}{{p} (S)} h ({p} _{S _d ^v})\nonumber \\&~\ge ~ h ({p} _S) - h \!\left( \sum _{v \in [\nu _d ]} \frac{{p} (S _d ^v)}{{p} (S)} {p} _{S _d ^v}\right) \nonumber \\&~=~ h ({p} _S) - h ({p} _S) \nonumber \\&~=~ 0 , \end{aligned}$$

where $${p} _S $$ denotes the class distribution vector of $$S$$, and Inequality (B1) is by concavity.

## Appendix C Further experimental results for $\hbox {CART}$

See Figs. 8 and 9.

## Appendix D Further experimental results on tree size

See Figs. 10 and 11.

## Appendix E Further statistical tests: pairwise Nemenyi

See Figs. 12 and 13.

## Appendix F Running time

Note that algorithm $\hbox {BAL}$ misses results over some datasets because its running time is too long Fig. 14.

All experiments were carried out on a server equipped with 24 processors of AMD Opteron(tm) Processor 6172 (2.1 GHz), 62GB RAM, running Linux 2.6.32$-$754.35.1.el6.x86_64. We use Python 3.8.5.

We also demonstrate the running time for two selected datasets with unit costs, with a large number of data objects and features, to explore the impact of number of objects and features to the running time. In general, as reflected in the worst-case time complexity $\mathcal {O} (H m n)$, the algorithms complete their computation quickly in the case of a large number of data objects ($n$), *or* large number of features ($m$), but not both. Furthermore, the dependency on $n$ is slightly worse than on $m$, as the tree height *H* typically has a logarithmic dependence on $n$ (Fig. 15).

## Appendix G Visual examples of real-life datasets

We visualize datasets that have meaningful features and whose trees are small enough to be contained in the paper. We also adjust the minimum leaf size (1% of the data size) to produce smaller trees.

### Visualization of decision trees for shoppers dataset

See Figs. 16, 17 and 18.

### Visualization of decision trees for breast-w dataset

See Figs. 19, 20 and 21.

### Visualization of decision trees for obesity dataset

See Fig. 22, 23 and 24.

### Visualization of decision trees for spambase dataset

See Figs. 25, 26 and 27.

### Visualization of decision trees for speed-dating dataset

See Figs. 28, 29 and 30.

## Footnotes

1 https://github.com/Guangyi-Zhang/low-expected-cost-decision-trees.

2 https://github.com/Guangyi-Zhang/low-expected-cost-decision-trees.

## Code Availability

Our implementation is publicly available in the Github repository (https://github.com/Guangyi-Zhang/low-expected-cost-decision-trees).
